# Amazonian drought of 2023: Environmental conditions relevant to fishes

**DOI:** 10.1111/jfb.70313

**Published:** 2026-01-23

**Authors:** Ora E. Johannsson, Thiago L. Nascimento, Helen Agasild, Priit Zingel, Rafael M. Duarte, Gudrun de Boeck, Anne Cremazy, Jhonatan Mota da Silva, Carolyn Morris, Chris M. Wood, Adalberto L. Val

**Affiliations:** ^1^ Department of Zoology University of British Columbia Vancouver British Columbia Canada; ^2^ Laboratory of Ecophysiology and Molecular Evolution, Brazilian National Institute for Research of the Amazon, INPA Manaus Brazil; ^3^ Department of Hydrobiology and Fisheries Institute of Agricultural and Environmental Sciences, Estonian University of Life Sciences Tartu Estonia; ^4^ Biosciences Institute, São Paulo State University‐UNESP, Coastal Campus São Vicente Brazil; ^5^ ECOSPHERE, University of Antwerp, Campus Groenenborger Antwerp Belgium; ^6^ Institut National de la Recherche Scientifique, Centre Eau Terre Environnement Québec Québec Canada; ^7^ Department of Biology McMaster University Hamilton Ontario Canada

**Keywords:** dissolved organic carbon, micro‐ and metazooplankton, Rio Negro, Rio Solimões, total suspended solids

## Abstract

This paper provides a platform for the following studies within this Special Issue. ‘Ecophysiology of fishes in the two great tributaries of the Amazon in the Anthropocene’. It documents the water quality conditions and accompanying zooplankton community structure and biomass relative to fish health in the Rio Negro and Rio Solimões during the extreme drought of 2023. Our goals were to document conditions, compare them with historical data and assess their implications for the resident fishes. During the 2023 drought, fishes in the Rio Negro came under more pressure from environmental changes than fishes in the Rio Solimões. Habitats became smaller and less diverse than normally observed by the end of the annual drought in both rivers. In some areas, fishes experienced elevated temperatures and/or reduced oxygen especially in the floodplain lakes and shallower areas. In the Rio Negro, increases in total suspended solids (TSS) decreased the range of vision for resident fishes, which would have both protected them from predators and shielded their prey. The coincidence of higher temperatures and higher oxygen in the afternoon in the Rio Negro suggests that primary production still occurred in the shallower, lit surface waters. This would explain the presence of zooplankton near the water's surface. Predation was likely intense as the macro‐zooplankton were small, although, the effect of high temperatures on metabolic rates could also influence size. Cladoceran biomass showed a negative relationship with the mass of the 1.2−50 μm fraction of TSS. Higher TSS concentrations likely meant resuspension of sediments. Spectroscopic analysis showed that dissolved organic carbon (DOC) was more degraded than normal, and thus would have provided less protection against the effects of low pH on ionoregulation. Low pH and low conductivity put extreme stress on maintaining ion balance in the black waters. In the Rio Solimões, fish suffered less environmental change. Water temperature and oxygen concentrations were more moderate, although evidence suggests that temperature and conductivity have increased compared to historic data. The concentration of TSS was normal. The greatest change was a 200% to 300% increase in DOC, albeit the DOC was very degraded. This may have spurred the high biomass of bacteria which was out of balance with the other components of the microbial loop. The passage of energy from bacteria to higher zooplankton trophic levels worked efficiently in the Rio Negro but not the Rio Solimões. This study allows a better understanding of the stresses on the fish community in these adverse conditions and the implications for their future.

## 1 INTRODUCTION

The Amazon basin ecosystem, with its extensive biodiversity, is the result of its ancient geology and geological history, its tropical location, its annual hydrological cycles (Cracraft et al., 2020; Val et al., 2022; Val & Almeida‐Val, 1995) and its age, which has allowed the evolution of biota to live in its diverse environmental conditions. Fish diversity is extensive, with 2716 species (1696 are endemic) representing 529 genera from 18 taxonomic orders (Dagosta & De Pinna, 2019; Val & Wood, 2022). The scales of the rivers and extent of their biodiversity are large. The principal river, the Amazon, runs from the foothills of the Andes Mountains to the Atlantic Ocean. In the upper regions, it is known as the Rio Solimões until it is joined by the Rio Negro just below Manaus, becoming the Amazon River. The studies in this Special Issue focus on fish living in the lower reaches of the Rio Negro and Rio Solimões.

The Rio Solimões is a white‐water river. The designation comes from the suspended clay which it transports from the foothills of the Andes (Wallace 1853). The river is 1600 km in length with an average floodplain width of 80 km (Encyclopedia Britannica, accessed 22 July 2025). Flow rate is 0.5–1.0 m s^−1^ during the dry period and twice of that during the wet season (Sioli, 1984; Val & Almeida‐Val, 1995). Average discharge was 102,458 m^3^ s^−1^ between 1973 and 2012 at Manacapuru, ~75 km upstream of the confluence with the Rio Negro (Filizola et al., 2009; Park & Latrubesse, 2015). Dagosta and De Pinna (2019) list a total of 922 species resident in the Rio Solimões. Várzea lakes, associated with white‐water systems, can be very productive (Fisher Jr. & Parsley, 1979; Val & Almeida‐Val, 1995) and have provided >90% of total fish yield of Amazonian inland fisheries (Petrere Jr., 1983 in Val & Almeida‐Val, 1995). These high levels of fish production come not only from the lakes themselves but are augmented by the forest when it is flooded. Leaves, seeds and fruits from the forest, as well as terrestrial arthropods, are important energy and protein sources for fishes in all water types (Correa & Winemiller, 2018). Although macrophyte and algal production can be important in Várzea lakes, all water types in the Amazon are heterotrophic (Richey et al., 1990; Val & Almeida‐Val, 1995).

The Rio Negro is the longest black‐water river in the world at 2250 km (Goulding et al., 2003 in Beltrão et al., 2019), with a drainage area of 696,808 km^2^ and an average discharge of 28,000 m^3^ s^−1^ (Goulding et al., 2003 in Beltrão et al., 2019). It is the fifth‐largest river in the world by water volume (Goulding et al., 2003 in Beltrão et al., 2019). The black colour comes from the lignin‐rich, allochthonous dissolved organic carbon (DOC) in the water, which sets the low pH characteristic of these systems (Wallace, 1853). pH values of 3.7 to 5.4 are common in the Rio Negro (Johannsson et al., 2017), which would normally be hazardous to fishes. Allochthonous DOC helps to protect fishes from excessive ion loss in these low‐pH, low‐ion‐concentration waters (Galvez et al., 2008; Morris et al., 2021; Wood et al., 2003). The dark colour limits light penetration into the water. The reduced depth available for photosynthesis, low‐nutrient concentrations and, in the main river, deep mixing contribute to the low rates of photosynthesis in black waters (Fisher, 1979). DOC molecules absorb the kinetic energy of light and release it, warming the adjacent waters, helping to create shallower, warmer epilimnia with cooler hypolimnia in black‐water lakes (Aprile & Darwich, 2009; Read & Rose, 2013). The Rio Negro supports the richest freshwater ichthyofauna in the world with 1165 species covering 56 families in 17 orders (Beltrão et al., 2019). Understanding the relationships between the ichthyofauna of both rivers and their environment is paramount to conservation efforts.

The productivity and habitat diversity of the river systems are dependent on the annual hydrological cycle, which can alter water depth in the rivers by approximately 10 m each year (Junk et al., 1989). Low water occurs in October in the Rio Negro and November–December in the Rio Solimões. In the region of Manaus, the heavy rains start in December–January, increasing through the early months of the year. Water fills the river channels and connecting lakes, overflows the banks and spills through the forest. One hundred and twenty thousand square kilometres of the Amazon basin are inundated (Myster, 2013). The rate of water decline is reduced by the austral rains. At low water, the principal habitats of fishes are the lakes, or what remains of them, the channels connecting lake habitat to the rivers and the main river itself. The fish community distributions expand and contract with the rhythm of the water. The best estimate of fish community size and composition is obtained during the low‐water period. Examination of the fish communities in Lake Janauacá (Rio Solimões) and Lake Prato (Rio Negro) at low water revealed two overall functionally similar communities dominated by piscivore biomass (41%–45%) followed by omnivore (27.4%–30%), and invertivore biomass (13.7%–18.4%) (Agasild et al., 2026, estimated from Figure 2, in this Special Issue). The percentages are for Lake Janauacá and Lake Prato, respectively. The remaining fish were divided among herbivores, detritivores and planktivores, all of which constituted less biomass in Lake Prato. For an understanding of the composition of the fish communities in the Rio Negro, consult Beltrão et al. (2019) and references therein; for the Rio Solimões, consult Dagosta and De Pinna (2019). In addition, Bogotá‐Gregory et al. (2020) provide an excellent comparison of fish community composition and structure between black‐ and white‐water Amazonian systems.

Extreme droughts are becoming more common in the Amazon (Marengo & Espinoza, 2016) and can have strong impacts on the fish communities. Through the alteration of water quality (temperature, oxygen etc), water flow rates and hydroconnectivity, drought alters fish distributions, behaviours and levels of stress (Röpke et al., 2017; Whitney et al., 2016). The resultant physiological challenges can result in reduced growth and reproduction, increase in disease, lower fitness and higher mortality rates (Hakala & Hartman, 2004; Lennox et al., 2019, a review). In the Amazon, droughts have been associated with increases in fish mortality, reductions in fish size and disappearance of some species (Guerreiro et al., 2016). Changes in community structure have been observed in the year following drought, with increases in planktivores, herbivores and detritivores coupled with decreases in omnivores and carnivores (Freitas et al., 2013).

The worst droughts ever recorded in the Amazon occurred in September–December 2023 and 2024, driven by higher temperatures associated with climate change and by the lack of austral rains characteristic of a strong El Niño system (Espinoza et al., 2024; Fernside & Araújo Silva, 2023). Massive fish and dolphin kills were reported as temperatures climbed and oxygen declined (Braz‐Mota & Val, 2024; Espinoza et al., 2024; Thomson, 2023). A scientific expedition scheduled for late November‐early December 2023 became an opportunity to study the physiology and habitat conditions of fishes in the Rio Negro (RN) and Rio Solimões (RS) under these extreme conditions. The resulting studies published in this Special Issue have focused on the effects of drought conditions on the physiology of fishes and changes in community structure.

This first paper within the Special Issue provides information on ecosystem conditions, particularly water quality within the study areas. Additionally, information on food resources (DOC, heterotrophic food web and surface zooplankton) is presented. The relevance of noteworthy changes in these conditions to the fish community is considered. The aims of this paper were to assess changes in ecosystem conditions compared to other low‐water periods, examine their implications for the well‐being of Amazon fishes and provide a background for the studies included in this Special Issue.

## 2 METHODS AND MATERIALS

### 2.1 Study areas and variables

**FIGURE 1:**
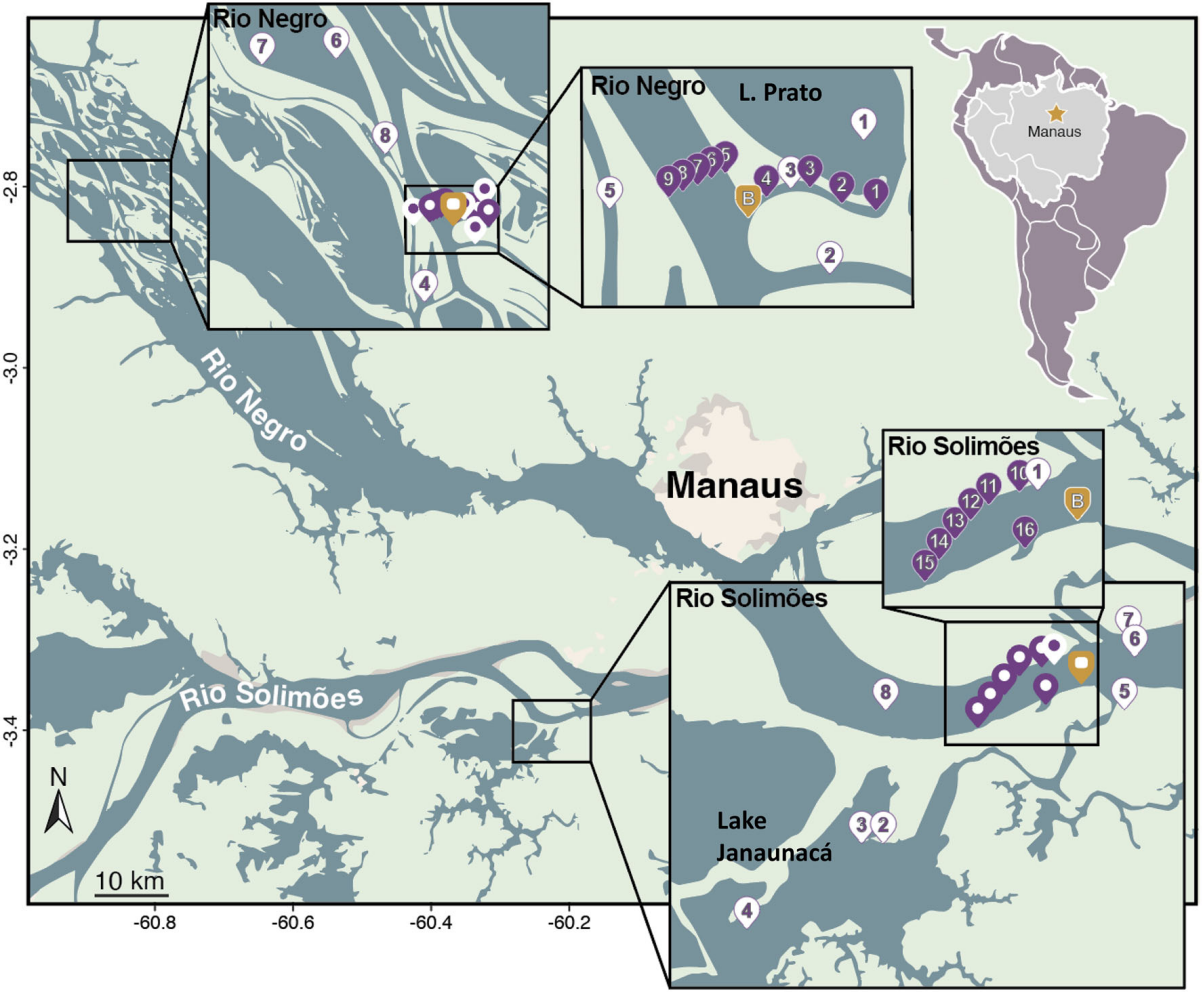
Map of the rivers and sampling locations within the Rio Negro and Rio Solimões in the state of Amazonas, Brazil. Purple way‐points numbered 1 to 16 were sampled at 5 cm depth. They are designated as S1–S16 in the text. White way‐points were sampled at the depth where fish were collected, usually 150 cm. They are designated in the text as PN1–PN8 for the Rio Negro sites and PS1–PS8 for the Rio Solimões sites. The yellow‐brown way‐point is the location of the ship. The station ID numbers, latitudes and longitudes, sampling depth and habitat conditions are provided in Table S2a,b.

The Rio Negro and Rio Solimões were each visited for 6 days at the height of the 2023 drought. The expedition was run from a ship, the Ana Clara, which was docked beside the ranger station Chico Mendes Institute for Biodiversity Conservation (ICMBio) (02^o^43′10.0′ S, 060^o^45′20.1″ W) in the Anavilhanus Archipelago in the Rio Negro between 23 and 28 November, and was anchored offshore of Manaquiri city (near Lake Janauacá) in the Rio Solimões (03^o^21′08.4″ S, 060^o^11′2.7″ W) from 29 November to 5 December (Figure 1). Small boats were used for sampling away from the ship. Lakes Prato and Janauacá had lost much of their water, shifting some studies to the connecting channels and rivers.

We examined water quality conditions (pH, conductivity, temperature and oxygen) at deep stations, where fishes were collected for many of the experiments reported in this Special Issue, as well as at surface sites (5 cm depth) in both rivers. Total suspended solids (TSS) were assessed at the surface sites. We measured components of the food web leading to planktivores, detritivores and omnivores [bacteria, heterotrophic nanoflagellates (HNF) and ciliates in the water column, as well as DOC, rotifers, cyclopoids, calanoids and cladocerans in near‐surface waters]. DOC was measured and analysed spectrophotometrically to determine specific absorbance indices and fluorescence components, which relate to fish health and the state, origin and functioning of the DOC (Table S1). No fishes were sampled for this study.

### 2.2 Sample collection and analysis|

#### 2.2.1 Site measurements and sample collection

Deep sites were located at a minimum distance of 2 m from the riverbank, at depths ranging from 40 to 150 cm in the water column (Figure 1). Habitats included lakes Prato and Janauacá, channels leading from these lakes to the larger river channels and the river itself. Temperature, conductivity and oxygen were measured using a YSI ProSolo meter (YSI Incorporated, Yellow Springs, Ohio, USA), and pH was measured using a YSI pH10A. Location was determined using a Garmin gps meter (Olathe, Kansas, USA) (Table S2a).

The surface samples (labelled S in the text) were collected at nine sites in the Rio Negro (S1–S9) and seven sites in the Rio Solimões (S10–S16), with S15 collected just off houseboats at the river's edge (Figure 1). Transects (S5–S9) and (S10–S15) ran across the rivers and S4 to S1 sampled the channel leading to Lake Prato, whereas S16 was in a channel leading towards Lake Janauacá. An additional sample (deep) was collected half a metre off the bottom in the middle of each transect (Table S2b). The Rio Negro was sampled between 9:30 AM and 2:45 PM on 23 November and the Rio Solimões on 30 November during the afternoon. After determination of the Secchi depth, temperature and oxygen were measured using a YSI probe, model 55. Five, 20‐mL water replicates were collected for DOC using 0.45‐μm polyethersulfone (PES) syringe filters (Hangzhou Cobetter Filtration Equipment Co., Ltd., Hangzhou, China). The syringes were immediately emptied into acid‐washed glass vials. These were held in the dark at <4°C prior to analysis. Water from 5‐cm depth (5 L) was brought back to the boat, where conductivity and pH were measured using a WTW 3310 conductivity meter (Xylem Analytics, Welheim, Germany) and a SympHony SP70P pH meter with a (C03243) pH probe (VWR International, Radnor, PA, USA).

#### 2.2.2 Diel patterns

Braz‐Mota et al. (2026 in this Special Issue) recorded temperature at 30 cm and at 3.0 m depths, every 10 min for 4–6 days using Pendant MX2201 temperature dataloggers (HOBO). The recorders were set up adjacent to the ranger station (ICMBios) in the Rio Negro from 22 to 28 November 2023, and near the Ana Clara at Manaquiri city in the Rio Solimões from 30 November to 4 December. GPS co‐ordinates are given in Section 2.1. The individual data for each day in 2023 can be found in Braz‐Mota et al. (2025), allowing comparison with historical data in the present study. The data were averaged across days within each hour of the day for each river to produce a composite 24‐h graph of epilimnetic and hypolimnetic means ±1 standard error of the mean (SEM). The first reading of each hour was composited from each day.

In 5–7 December 2014, temperature, oxygen, pH and conductivity were recorded at 10 cm depth in the ICMBio complex (2^o^43′10.9″S, 60^o^45′18.8″W) using an HI 929828–1.3 multimeter, model HIOKI 9828 V2.1 (Hioki E.E. Corporation,81 Koizumi, Ueda‐shi, Nagano, Japan). This was used as the historical dataset, facilitating comparison with a year of normal low water.

#### 2.2.3 Total suspended solids

A portion of the 5 L of water collected at 5 cm depth was filtered on the ship for TSS. PES 1.2‐ and 0.45‐μm filters (polyethersolfone filters, Hangzhou Cobetter Filtration Equipment Co., Ltd., Hangzhou, China) were prepared prior to the trip. Filters were dried for 24 h at 65°C, placed in individual plastic, sealable Petri dishes in a desiccator and later weighed to 0.0001 g on a Sartorius CP2245 balance (Sartoriius Corp., Heartland Blvd., Edgewood, NY, USA). The filters were sealed in their individual Petri dishes and frozen. On the ship, the 5–l container of river water was shaken vigorously. Fifty to 100 mL was removed and passed through a 50‐μm Nitex mesh, positioned over the filtration cup of the filtering apparatus to remove larger objects, and then through the 1.2 and 0.45‐μm PES filters sequentially. The filters were resealed in the plastic Petri dishes and frozen. In Manaus, they were dried to constant mass and re‐weighed. The initial masses were subtracted from the final masses, corrected for the volumes filtered and converted to mg L^−1^ TSS. Three replicate pairs of filters (1.2 and 0.45 μm) were collected per station.

#### 2.2.4 DOC

DOC concentrations were measured using a high‐temperature Shimadzu TOC‐V_CSH_ total organic carbon (TOC) analyser (Shimadzu, Kyoto, Japan) set to non‐purgeable organic carbon (NPOC) processing. The machine was calibrated daily using standards prepared according to the manufacturer's manual. Ultrapure water blanks (MilliporeSigma, Burlington, MA, USA) were run at the beginning and end of each day. Absorbance and fluorescence profiles were measured on a Genesys 10S UV–Vis spectrophotometer (Daly City, CA, USA) within 1–4 days of collection. Ultrapure water profiles were subtracted from the measured absorbance and fluorescence data prior to analysis. Absorbance and fluorescence indices related to fish health and DOC processing were calculated. Abs_250‐550_, SUVA_254_, SAC_340_, SAC_Ka310_, *R*_254/365_, *S*_R_ and FI were calculated as described in Table S1.

#### 2.2.5 Microbial loop and metazoan zooplankton

Metazoan zooplankton were surveyed for abundance and biomass. At the time of the surface water quality sampling, 10 L of water was collected from 5 cm depth at each site and filtered through a 50‐μm mesh net. The retained zooplankton were preserved in 70% ethanol. The zooplankton (cladocerans, copepods and rotifers) were counted in a Bogorov chamber (Wildco, Yulee, FL, USA) under a Nikon AZ100 dissecting microscope at 80× magnification. The total volume of each sample was analysed. Abundance was recorded, and biomass was calculated based on size measurements and equations for Neotropical zooplankton. Zooplankton carbon biomasses were estimated by length‐dry mass regressions for copepods (Azevedo et al., 2012), cladocerans (Maia‐Barbosa & Bozelli, 2005) and copepod nauplii and rotifers (Dumont et al., 1975), using the carbon conversion factor of 0.48 mg C per mg dry mass (Andersen & Hessen, 1991).

The microbial loop community (bacteria, HNF, ciliates) was sampled in both rivers using a Ruttner sampler (KC‐Denmark Research Equipment, Silkeborg, Denmark) (stations S1–S3, and S12, S13 and S15). Integrated samples were created by pooling water collected at intervals of 0.5 m depth through the entire water column. From the integrated water sample, a 100‐mL subsample for ciliate analysis was fixed with acidic Lugol [4% Lugol's iodine (v/v)]. Subsamples (50 mL) were taken for bacteria and HNF analyses and immediately fixed by adding glutaraldehyde to a final concentration of 2% (v/v).

Ciliates were counted in sedimentation chambers under an inverted microscope (Nikon Eclipse Ti) at 600× magnification, following Utermohl (1958). Ciliate biovolumes were calculated from measurements of length and width dimensions of animals with approximations to an appropriate geometric shape (Zingel & Nõges, 2008). For conversion to carbon biomass, the factor 0.19 pg C μm^−3^ was used (Putt & Stoecker, 1989). Prior to analyses, the subsamples of bacteria and HNF were stained for 10 min with 4′6‐diamidino‐2‐phenylindole (DAPI) at a final concentration of 10 μg DAPI mL^−1^ (Porter & Feig, 1980) and filtered under low pressure (<0.2 bar) for counting onto 0.2‐ and 0.8‐μm pore size black Nuclepore filters (Whatman International Lt., Kent, UK), respectively. The abundances of bacteria and HNF were determined by direct counting of cells using epifluorescence microscopy (Nikon Eclipse Ti) at 1000× magnification. At least 400 bacteria cells from different fields were counted for each sample with a ultraviolet (UV) filter (420 nm). All specimens of HNF found within 1.6 mm^2^ were counted on each filter. The microscope was equipped with a pale‐yellow UV (420 nm) and a blue (515 nm) filter to distinguish heterotrophs from mixotrophs and autotrophs during HNF counting. Conversion to carbon biomass was made using a factor of 0.22 pg C μm^−3^ for bacteria and HNF (Borsheim & Bratback, 1987; Bratback & Dundas, 1984).

### 2.3 Data preparation and statistical analyses

#### 2.3.1 DOC

DOC concentrations were variable across the transects; however, ABS_250–550_ did not vary significantly across either river: Rio Negro (S4–S9, Coefficient of Variation (COV) 0.9%) and Rio Solimões (S10 and S14, COV <9%), where COV% = mean divided by the standard deviation (SD) × 100. Given the variability in both DOC datasets and the stability in the ABS_250–550_ data, the average DOCs for each river transect were used to calculate the DOC‐specific indices (SUVA_254_, SAC_340_ and SAC_Ka310_). Although ABS_250–550_ and DOC differed markedly from the transect means, the station mean DOC was used in the calculations. Index and DOC values for deep water (2.5 m in the Rio Negro and 5.0 m in the Rio Solimões) were not different from surface waters values in both rivers; therefore, the deep‐water data were included in all transect averages and statistical tests. In the Rio Solimões, only one or two replicate samples were analysed due to high demand for the spectrophotometer in the second week of the expedition. Therefore, statistical exploration of spatial patterns was not possible in this river, as was done for the Rio Negro using analysis of variance (ANOVA) analyses (see Section 2.3.3).

DOC absorbs light as it passes through the water in the river, removing UVB radiation in the surface waters. UVB is dangerous to both plants and animals. Given the presence of zooplankton at 5 cm depth, we asked if UVB exposure could be a problem. We calculated the proportion of UVB removed in the top 5 cm of water column. The absorbance profiles between 280 and 320 nm permitted calculation of μ_(ʎ)_ (http://www.physics.uoguelph.a/pgarrett/Teaching.html, 2016), using the following equation:

$$\mu_{\left(ʎ\right)}=\text{Absorbance}\left(ʎ\right)/0.4343\;\mathrm{at}\;d=1\;\mathrm{cm}$$

where *ʎ* is the wavelength and ‘*d*’ is the distance through the cuvette used to measure absorbance. Light penetration was calculated as an exponential function of depth, where

$$\text{Depth of light penetration}\;\left(ʎ\right)\;\left(1\%\right)=\left[\ln\left(100\right)-\ln\left(1\right)\right]/\mu_{\left(ʎ\right)}$$

#### 2.3.2 Zooplankton and microbial loop communities

HNF are a crucial link in the microbial loop. Therefore, it is important to understand the main mode of regulation of HNF abundance. For this purpose, we used Gasol's (1994) theoretical model to plot corresponding abundances of HNF and bacteria. According to Gasol's theory, data points located below the mean realized abundance (MRA) line suggest top‐down control on HNF. Points above the MRA line imply low top‐down control on HNF. Points that are close to the maximal attainable abundance (MAA) line point to strong bottom‐up control on HNF.

#### 2.3.3 Statistical analyses

All statistical analyses and graphing were performed using Graphpad PRISM 10.0 (Graphpad Software Inc., CA, USA). A *p* ≤ 0.05 was accepted as significant. All data analysed statistically were log transformed as some treatments did not have normal distributions, as assessed by the Shapiro–Wilk test or equal variances (*F* test for variance). Unpaired Student's *t*‐tests were used to compare (a) surface water quality conditions and (b) DOC concentrations and quality in the transects between the two rivers. The deeper water quality data, which had been recorded at the sites of fish collection, were compared across habitats using ANOVA followed by Tukey's multiple comparisons test. In the Rio Negro, the habitats were the lake and channels, whereas in the Rio Solimões, they were the channels and the river. Only one sample was collected in the Paraná; therefore, it was omitted. A potential relationship between cladoceran biomass and the mass of TSS in the size range of 1.2–50 μ was assessed using regression analysis. Heteroscedasticity of the residuals was tested using the Beusch–Pagan test. All data are presented as non‐logged averages ±1 SEM (N).

## 3 RESULTS

### 3.1 Water quality

**FIGURE 2:**
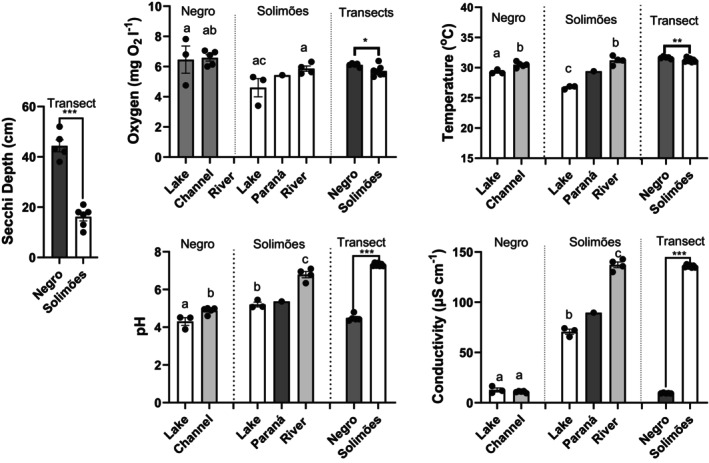
Comparison of water quality variables among habitats and between the Rio Negro (RN) (Anavilhanas Archipelago) and Rio Solimões (RS) during the November 2023 drought. The data in panels labelled ‘Negro’ and ‘Solimões’ were collected at depth, whereas the ‘transect’ data were collected subsurface at 5 cm depth across each river transect. See Figure 1 and Table S2a,b for station locations and descriptions. All data were log transformed for statistical analyses. Data are presented as means ±1 standard error of the mean (SEM). Dots are the non‐transformed data points. The ‘Negro’ and ‘Solimões’ data were compared within a single analysis of variance (ANOVA). Differences among habitats were assessed using Tukey's multiple comparisons tests, *n* = 7, 8 per river. Letters above the bars indicate which are significantly different. The ‘transect’ data were compared using Welch's log‐normal *t*‐test, *n* = 5, 7, except for the Secchi depth comparison where *n* = 5, 6. The level of significant difference is indicated by the number of *.

Water quality sampling and analyses revealed differences in Secchi depth, temperature, oxygen, pH and conductivity between the two rivers and among habitats (Figure 2a–e; Table S2a,b). The Rio Solimões paraná data (Figure 2) were excluded from data analysis because they were intermediate between lake and river values, reflecting a mixing of the two waters as river water began moving up the channel. With respect to the deeper samples (1) lake temperatures were significantly lower than the temperatures in the main channels of both rivers (Figure 2c). In the Rio Negro, lake included sampling sites PN6 and PN7, which were located in a much‐widened area of the river channel which narrowed to a small outlet (Figure 1). (2) In the Rio Solimões, pH and conductivity were also significantly lower in Lake Janauacá than in the main river (Figure 2d,e). (3) Conductivity and pH were significantly lower in the Rio Negro than in the Rio Solimões while oxygen and temperature were slightly higher (Figure S2b–e). (4) Transparency, as measured by Secchi depth, was higher in the Rio Negro than in the Rio Solimões (Figure 2a).

**FIGURE 3:**
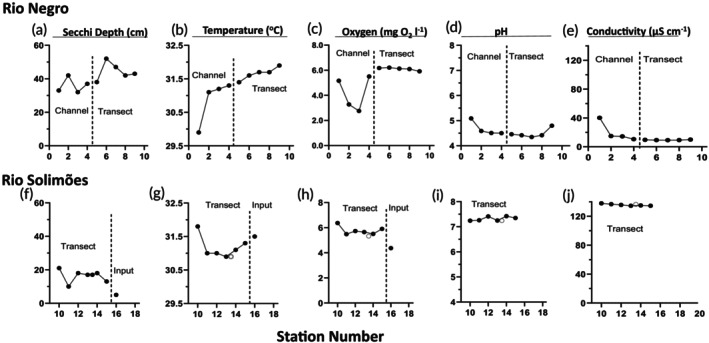
Subsurface (5 cm depth) water quality data from the Rio Negro and Rio Solimões in November 2023. In the Rio Negro, sites were located down a channel which had been attached to Lake Preto (S1–S4), and along a transect across the connecting branch of the river (S5–S9) (see Figure 1). In the Rio Solimões, the transect goes along one shore (S10 and S11) and then diagonals across the river to the river boats (S12–S15). S16 is in an input channel. Each RS data point represents one measurement. Rio Negro: (a) Secchi depth, (b) temperature, (c) oxygen, (d) pH and (e) conductivity. Weather prevented collecting water quality data from the deep sample. Rio Solimões: (f) Secchi depth, (g) temperature, (h) oxygen, (i) pH and (j) conductivity. Deep sample data are represented by open circles.

Spatial patterns in the surface water quality data reveal shifts in all measures within the channel leading to Lake Prato in the Rio Negro (Figure 3a–e), with decreases in Secchi depth, temperature and oxygen, and increases in pH and conductivity. S1, the station in the channel closest to Lake Prato, was shallow and areas were on the verge of drying up. The patterns up the channel from the river to S1 showed increases in conductivity, most markedly at S1 (Figure 3e). Oxygen was low at S2 and S3 but increased at S1 (Figure 3c). pH climbed slowly from S4 to S2 then markedly at S1 (Figure 3d). Temperature fell slowly along the channel and then markedly decreased at S1 (Figure 3b). Secchi depths were erratic but generally shorter in the channel than in the river (Figure 3a). Within the Rio Solimões, river edge effects are observed in Secchi depth, temperature and oxygen across the river transect (Figure 3f–h); pH and conductivity remain constant.

### 3.2 Diel patterns in temperature and oxygen

**FIGURE 4:**
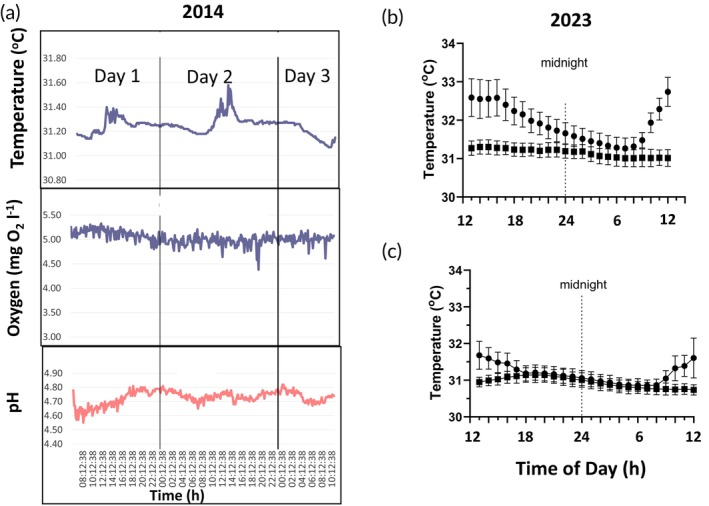
Diel patterns in water quality patterns. (a) patterns in temperature, oxygen and pH between 7 and 9 December 2014 at the Rio Negro Ranger Station moorage (Figure 1). Data were collected at 10 cm depth and at 10‐min intervals. (b) In 2023, patterns in surface (●) (10 cm depth) and near‐bottom (■) (3.0 m depth) temperatures were collected at 10‐min intervals over a period of 6 days while at the moorage in the Anavilhanas Archipelago (Rio Negro) (Figure 1). (c) A similar set of data were collected over 4 days at the boat moorage near Manaquiri City in the Rio Solimões (Figure 1). In b and c, data from the start of each hour were averaged, starting at 13:00 PM. Means ± 1 standard error of the mean (SEM) (*n* = 6 or 4).

In December 2014, at ICMBio in the Rio Negro, diel cycles were observed in temperature and pH but not oxygen at 10 cm depth (Figure 4a). Temperature peaked in the early‐mid‐afternoon, whereas the highest pHs occurred overnight. The temperature cycles were greatly muted compared to those in 2023 (Figure 4b,c). Conductivity (not shown) was constant at 8 μS cm^−1^.

Surface temperatures during November–December 2023, collected at 10 cm and 3.0 m depths at the ICMBio station in the Rio Negro and near Manaquiri City in the Rio Solimões (Braz‐Mota et al., 2026, in this Special Issue), also cycled dielly. Peak temperatures occurred in the afternoon, principally between 13:00 and 16:00 PM, and ranged in intensity: 5 out of 6 days had peaks >33.0°C in the Rio Negro. The peaks varied in intensity and were not well captured when data were averaged (Figure 4b). Peaks were more intense in the Rio Negro than in the Rio Solimões (Figure 4b,c). The oscillations in temperatures at depth (3.0 m depth) were dampened. The daily length of time of overlap between surface and deep temperatures was more extensive in the Rio Solimões (from 17:00 PM to 09:00 AM) than in the Rio Negro (from 23:00 PM to 09:00 AM), where surface waters reached higher temperatures and required longer to cool. Braz‐Mota et al. (2026, in this Special Issue) saw no clear diel patterns in oxygen in either river.

### 3.3 Suspended sediments

**FIGURE 5:**
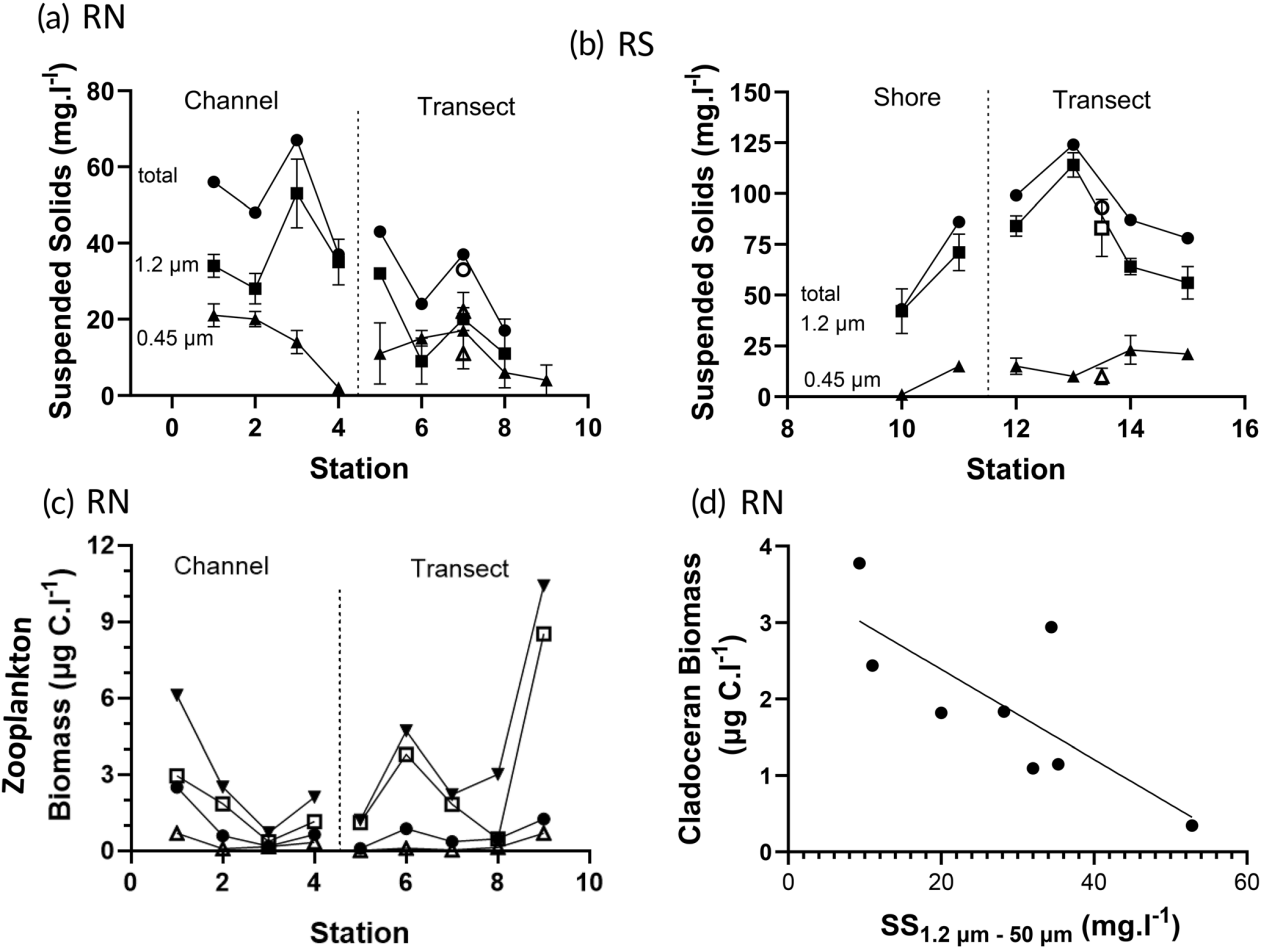
Distribution of suspended solids (SS) by size fractions measured during drought conditions of 2023: 50–1.2 μm () 1.2–0.45 μm filter (▲) and total suspended solids (TSS) (●) from samples collected at 5 cm depth along a transect and into a channel (a) in the Anavilhanas Archipelago, Rio Negro (RN) and (b) in the Rio Solimões (RS) near Janauacá village. Means ± 1 standard error of the mean (SEM) (*N* = 1 to 3). The mass of SS components for the deep station in each river has open symbols. (c) Rio Negro near‐surface metazooplankton biomass across stations: total (■), cladocerans (□), copepods (●) and rotifers (Δ). (d) Correlative relationship between cladoceran biomass and mean SS: 50 μm – 1.2 μm mass (SS_50–1.2μm_) in Rio Negro surface samples: S9 SS_50–1.2μm_ data were not available. Cladoceran biomass = −58.88 × SS_50–1.2μm_ + 3.568, *p* = 0.0278, *R* ^2^ = 0.5805, *N* = 8. The Beusch–Pagan test of residuals found no significant heteroscedasticity.

TSS were less abundant in the transect across the Rio Negro (Figure 5a) than across the Rio Solimões (Figure 5b). In the Rio Solimões, TSS peaked in mid‐stream at 124 mg L^−1^, falling to 78 mg L^−1^ towards the ends of the transect. The transect average TSS was 95 ± 6 mg L^−1^ (*n* = 8). TSS at depth was similar in mass to that at the surface, 93 mg L^−1^ (Figure 5b). The portion retained in the larger size range (50–1.2 μm) varied from 67% to 100%.

In the Rio Negro, the same pattern was developing across the transect from S8 to S5–S6 (no S9 data). TSS rose across the transect from levels of 17.3 mg L^−1^ at station S8 to 24.3 mgL^−1^ at S6. However, TSS did not decline at S5 and S4 but increased in the 50–1.2 μm particle size range. TSS were even higher in the channel (stations S1–S3), with values ranging from 47.9 to 67.2 mg L^−1^ (Figure 5a). Again, more mass was observed in the larger size range (50–1.2 μm), especially at station S4. Percentage contribution of the 50–1.2 μm size range ran from 54% to 96% from S1 to S3. Only at station S6 was more mass collected in the 0.45–1.2 μm size range. The TSS collected at depth and at the surface at station S7 were similar in mass, 33 versus 37 mg L^−1^ (Figure 5a).

Cladoceran biomass was a mirror image of TSS mass across the Rio Negro sampling sites (Figure 5a,c). Cladocerans are usually filter‐feeders (Hessen, 1985). The negative relationship between TSS mass in the 50–1.2 μm range and cladoceran biomass was assessed using regression analysis and found to be significant (Figure 5d). The residuals were homoscedastic.

### 3.4 Dissolved organic carbon

**FIGURE 6:**
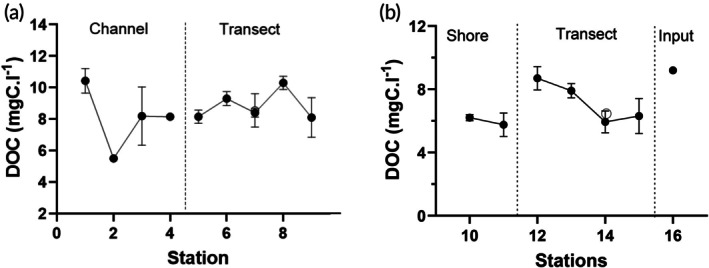
Dissolved organic carbon concentrations in (a) the Rio Negro (RN) and (b) the Rio Solimões (RS). Samples were collected at 5 cm depth along the transects (S5–S9) and (S10–S15) on the RN and RS, respectively, and along the channel (S1–to S4) in the RN and an input channel in the RS (S16). Means ±1 standard error of the mean (SEM) (*N* = 2 or 3).

DOC and all its indices were significantly different between the two river transects (Table 1). The mean DOC in the Rio Negro was 8.7 mg C L^−1^ and 6.7 mg C L^−1^ in the Rio Solimões. In the Rio Negro, DOC concentration was variable but more variable within the channel than across the transect (Figure 6a). In the Rio Solimões, DOC peaked in the middle of the transect at stations S12 and S13 (Figure 6b). At S16, in an input channel, DOC rose to 9.2 mg C L^−1^ (*N* = 1). Based on the calculations of absorption of light by DOC as it passes through the water in the rivers, DOC removed 99% of UVB radiation by 5 cm depth in the Rio Negro and could have removed 50% UVB radiation in the Rio Solimões in the absence of the suspended clay particles.

**TABLE 1:** Dissolved organic carbon (DOC) properties. Average DOC concentrations and spectrophotometric and fluorescence indices from the Rio Negro transect and Rio Solimões transect and two additional habitats.

River	Rio Negro		Rio Solimões		
Stations (*N*)	Transect (S4–S9, 7D) (21)	ANOVA between transects	Transect (S10–S14, 13D) (8)^a^	Near boats (S15) (1)	Input (S16) (1)
DOC (mg C L^−1^)	8.7 ± 0.31 (7)	*p* = 0.0231	6.9 ± 0.51 (6)	6.3 ± 0.91 (3)	9.2 (1)
Absorbance indices					
ABS_250–550_ DOC^−1^	0.3624 ± 0.0012	*p* < 0.0001	0.1096 ± 0.0068	0.2199	0.0495
SUVA_254_	4.025 ± 0.0159	*p* < 0.0001	1.470 ± 0.0932	1.976	0.7385
SAC_340_	34.11 ± 0.1226	*p* < 0.0001	8.307 ± 0.5364	16.53	3.20
SAC_Ka310_	4.992 ± 0.0188	*p* < 0.0001	1.518 ± 0.0953	2.485	0.668
*R*_254/365_	3.857 ± 0.0039	*p* < 0.0001	5.7500 ± 0.1148	3.389	7.591
Slope ratio (*S*_R_)	0.7516 ± 0.0034	*p* < 0.0001	0.9827 ± 0.0362	1.535	1.048
Fluorescence indices					
FI	1.355 ± 0.0074	*p* < 0.0001	1.5906 ± 0.0100	1.4863	1.7959

*Notes:* Means ± 1 SEM (*N*) are given with each station. The deep station data (7D, 13.5D) were included with the surface transect data as they were not significantly different. The mean transect DOCs were used to calculate the DOC‐specific spectrophotometric indices. Analysis of variance (ANOVA) of the indices across stations had indicated that there were no significant differences within the defined transects; therefore, all replicates were included in each variable analysis. Indices were compared between transects using unpaired *t*‐tests of the logged data.

^a^ Availability of the spectrophotometer was limited by the time we were working in the RS.

**FIGURE 7:**
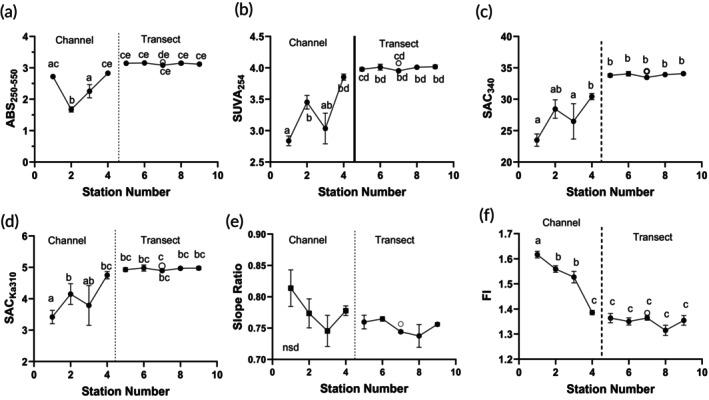
Dissolved organic carbon (DOC) spectral characteristics from the Rio Negro. DOC samples were collected from 5 cm depth at stations S1 to S9 (●) and at depth at station 7 (O), 23 November 2023. Means ± 1 standard error of the mean (SEM) (*N* = 3). Spectral indices were tested for differences among the sites using analysis of variance (ANOVA) and Tukey's multiple comparisons tests of the logged data. Different letters above the points indicate differences among stations. (a) Absorbance_250–550_, (b) SUVA_254_, (c) SAC_340_, (d) SAC_Ka310_, (e) slope ratio (*S*_R_) and (f) fluorescence index (FI). For descriptions of the indices, see Table S1.

In the Rio Negro, DOC absorbance and fluorescence indices were relatively stable across the transect from S9 to S4, including the deep‐water sample (Figure 7a–f). Notably, all the indices related to specific absorbance; that is, an absorbance measure divided by DOC, declined along the channel reaching lowest levels at S1 (Figure 7b–d), whereas the slope ratio (*S*_R_) and fluorescence index (FI) increased (Figure 7e,f). No significant differences in the mean molecular size of DOC were observed as indicated by a lack of change in the ratio *R*_254/365_ (Figure S1a). The FI averaged 1.359 ± 0.009 (*n* = 7) between S4 and S9 (Figure 7f), but it was >1.5 at the S3–S1 channel stations.

**FIGURE 8:**
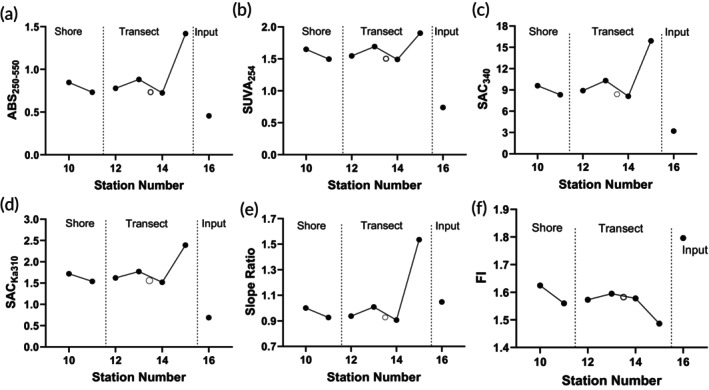
Spectral characteristics of dissolved organic carbon (DOC) from the Rio Solimões. Samples were collected 5 cm below the surface at sites S10 to S16 on 30 November 2023. Deep samples, collected between S13 and S14, are presented as open circles (*N* = 1). (a) ABS_250–550_, (b) SUVA254, (c) SAC_340_, (d) SAC_Ka310_, (e) slope ratio (*S*_R_) and (f) fluorescence index (FI). For descriptions of the indices, see Table S1.

In the Rio Solimões, absorbance indices and FI were relatively constant from the nearshore at S10 to the third transect station, S14, including the data from the deep samples (Figure 8a–f). All indices were higher in the Rio Negro than in the Rio Solimões, with the exception of *R*_254/365_, *S*_R_ and FI (Table 1). The absorbance indices, along with *S*_R_, increased at S15 just offshore of the houseboats – *S*_R_ rose from 0.9824 to 1.535. Mean molecular size (*R*_254/365_) decreased to 3.888 (Figure S1b), indicating an increase in mean molecular weight, and FI fell below 1.5. The indices had the opposite pattern in the input channel, S16.

### 3.5 Metazooplankton and microbial loop communities

Cladocerans dominated the metazooplankton abundance and biomass in the surface waters of the Rio Negro, primarily represented by three species: *Moina micrura*, *Bosmina hagmanni* and *Bosminopsis deitersi*. Table S3a,b provide information on metazoan zooplankton species abundance, biomass and distribution in the surface waters of the Rio Negro. Station S9, on the far side of the transect, had the highest metazooplankton biomass (Table S3b). Among copepods, the cyclopoid *Thermocyclops minutus* and their copepodites dominated. In the Rio Solimões, limited zooplankton were found in the near‐surface samples, with only one cladoceran and two copepod individuals.

**FIGURE 9:**
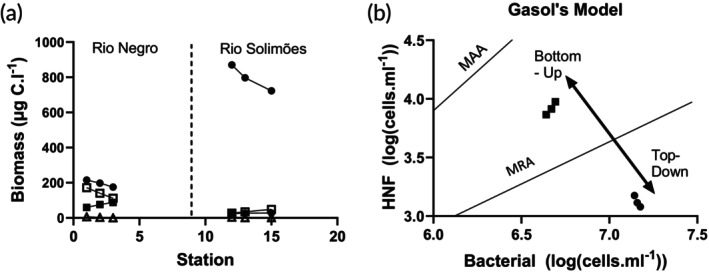
Micro‐ and metazooplankton biomass in the Rio Negro and Rio Solimões. (a) Biomass of zooplankton in integrated samples from the Prato channel (Rio Negro) (S1–S3) and transect in the Rio Solimões (S12, S13 and S15). Bacteria (●), heterotrophic nanoflagellates (HNF) (□) and ciliates (■) compared with metazoan zooplankton (Δ). (b) Bacterial and HNF abundance in Rio Negro and Rio Solimões plotted following Gasol's model (1994). MAA, the maximum attainable abundance line; MRA, the mean realized abundance line. Rio Solimões (●), Rio Negro (■).

Within the planktonic compartment analysed (bacteria, HNF, ciliates, metazoan zooplankton), the metazoan zooplankton had the lowest carbon biomass (Figure 9a). Bacteria had substantially higher abundance and biomass in Rio Solimões, whereas ciliates and HNF had higher abundance in the Rio Negro (Figure 9a; Table S4b). Plotting abundances of HNF and bacteria according to Gasol's (1994) model indicated opposite patterns of HNF regulation between the rivers: bottom‐up control in the Prato channel (RN) and strong top‐down control in Rio Solimoes (in the river) (Figure 9b).

## 4 DISCUSSION

These environmental data collected at the nadir of drought conditions in the Rio Negro and Rio Solimões found five important changes from previous low‐water periods, with relevance to fish well‐being. Table 2 provides a comparison of historical water quality variables and those documented in the present study. The most obvious and dramatic was the loss of freshwater habitat in both systems. For the Rio Negro, the next important change was the spike in afternoon temperatures in the flowing river channels, documented by Braz‐Mota et al. (2026, in this Special Issue). A major increase in TSS was the third problem in the Rio Negro (Figure 5a; Table 2). In the Rio Solimões, the second problem, after the loss of freshwater habitat, was the trophic inefficiency within the microbial food web and, therefore, the planktonic food web (Figure 9b). In both rivers, DOC was degraded compared to past low‐water years (Table 2). DOC performs a number of ‘services’ for fish and the ecosystem.

**TABLE 2:** Comparison of historical and present water quality variables.

Variable	Historical low‐water period	References	November–December 2023	References
			Severe drought	
			Low‐water period	
Rio Negro				
Time of minimum water level	Late October–early November		22 November 2023	Present study
Secchi depth (cm)	90–100	Johannsson et al. (2017)	32–52	Present study
Temperature (°C)	Surface LP 31.1–37.2	Johannsson et al. (2017)	ICMBio surface 30.33–35.18	Braz‐Mota et al. (2026)
	Deep LP 30.5–31.1	Present study	ICMBio deep 30.14–31.83	Braz‐Mota et al. personal communication^b^
	ICMBio 31.2–31.6	Zingel et al. (2026)	Prato channel^b^ 30.7–32.7	Present study
	River 30.1–32.4		Prato channel 31.2 (30.6–32.7)	Zingel et al. (2026)
	LP 2019 31.2 (30–32.3)		Deep channels 29.1–31.1	
			Surface river/channel 29.9–31.9	
Oxygen (mg O_2_ L^−1^)	ICMBio 4.95–5.23	Johannsson et al. (2017)	Prato channel 2.17–4.59	Braz‐Mota et al. (2026)
	Lake Prato >6	Present study	Deep river 6.00–7.05	Zingel et al. (2026)
	Main river 5.0–5.7	Zingel et al., 2026	Surface transect 5.90–6.21	Present study
	Main river 2.9–4.2		Surface channel 2.76–5.50	
	LP 2019 5.3 (4.1–6.6)		Prato channel 3.1 (2.2–4.8)	
pH	ICMBio 4.6–4.8	Johannsson et al. (2017)	Deep river 3.9–5.0	Present study
	Lake Prato 4.3–5.0	Zingel et al., 2026	Surface river 4.4–4.8	Zingel et al. (2026)
	Main river 3.9–4.5	Present study	Surface channel 4.5–5.1	
	LP 4.5 (4.3–4.9)		Prato channel 5.1 (4.8–5.4)	
Conductivity (μS cm^−1^)	ICMBio 8.0	Present study	Deep river 10.6–12.3	Present study
			Surface river 9.6–10.0 Surface channel 10.5–40.2	
TSS (mg L^−1^)	Usually <5 mg L^−1^ and always <11 mg L^−1^ 8 mg L^−1^ (2015)	Crémazy et al. (2019)	Surface river 17.0–43.0	Present study
		Marinho and Cremon (2020)	Surface channel 36.8–67.2	
DOC (mg C L^−1^)	Lake Prato 8.1–8.7	Johannsson et al. (2017)	Surface river 8.7	Present study
	Main river 9.9–12.2 7–15	Johannsson and Holland (2024)	Surface channel 5.8–10.4	
DOC‐ABS_250–550_ DOC^−1^ (cm^2^.mg^−1^)	Lake Prato 0.787–0.813	Johannsson et al. (2017)	Surface river 0.3624	Present study
	Main river 0.814–0.821		Surface channel^a^ 0.3472–0.2617	
DOC‐SUVA_254_ (cm^2^ mg^−1^)	Rio Negro 5.0–7.4	Johannsson et al. (2017)	Surface river 4.025	Present study
		Johannsson and Holland (2024)	Surface channel^a^ 3.608–2.924	
DOC‐SAC_340_ (cm^2^ mg^−1^)	Lake Prato 45–48	Johannsson et al. (2017)	Surface river 34.11	Present study
	Main river 44–45	Johannsson and Holland (2024)	Surface channel^a^ 30.43–24.62	
	Rio Negro 39–73	Holland et al. (2017)		
DOC‐SAC_Ka310_	Lake Prato 6.941 (2014)	Johannsson (personal communication)	Surface river 4.992	Present study
	Main river 6.768 (2014)			
DOC‐*R*_254/365_	Lake Prato 3.67–3.80	Johannsson et al. (2017)	Surface river 3.8570	Present study
	Main river 3.70–3.78 Rio Nego 3.43		Surface channel^a^ 3.846–3.794	
DOC‐*S*_R_			Surface river 0.7516	Present study
			Surface channel^a^ 0.778–0.787	
DOC‐FI	Rio Negro 1.37	Johannsson et al. (2017)	Surface river 1.355	Present study
	Lake Prato 1.02–1.10	Holland et al. (2017)	Surface channel^a^ 1.386–1.617	
	Main river 1.05–1.12			
Rio Solimões				
Time of minimum water level	Late November–early December		Late November–early December	
Secchi depth (cm)	2–20	Brito et al. (2016)	10–21	Present study
Temperature (°C)	29.0 (<1984), 30.1 by 2013 LJ. 30 (29.2–32.2)	Val and Almeida‐Val (1995)	River 50 cm 30.7–31.5	Braz‐Mota et al. (2026)
		Zingel et al. (2026)	River 10 cm 30.28–32.04	Present study
			River 300 cm 30.33–31.49	Braz‐Mota et al. personal communication
			Deep river 30.3–32.2	Zingel et al. (2026)
			Surface river 30.9–31.8	
			Deep lake 26.4–26.9	
			Channel 30.8 (30.5–31.8)	
Oxygen (mg O_2_ L^−1^)	LJ. 5.5 (5.2–6)	Zingel et al. (2026)	River 50 cm 4.78–5.59	Braz‐Mota et al. (2026)
			River deep 5.43–6.31	Present study
			Lake deep 3.40–5.11	Zingel et al. (2026)
			River surface 5.50–6.37	
			Channel 5.2 (4.8–5.6)	
pH	6–8 LJ. 6.5 (6.4–6.7)	Val and Almeida‐Val (1995)	Deep river 6.4–7.1	Present study
		Brito et al. (2016)	Deep lake 5.1–5.5	Souto et al. (2015)
		Zingel et al. (2026)	Surface 7.25–7.42	Zingel et al., 2026
			Channel 6.9 (6.8–6.9)	
Conductivity (μS cm^−1^)	57 (<1984), 71 (2013)	Furch (1984), Brito et al. (2016)	Surface river 134.5–137.8	Present study
TSS (mg L^−1^)	37–93 70 (2015)	Crémazy et al. (2019)	Surface river 48.8–143.9	Present study
DOC (mg C L^−1^)	2.5–2.8	Crémazy et al. (2016) Holland et al. (2017)	Surface river 6.9	Present study
DOC‐ABS_250–550_ DOC^−1^ (cm^2^ mg^−1^)			Surface river 0.1096	Present study
DOC SUVA_254_	7.32	Holland et al. (2017)	Surface river 1.4695	Present study
DOC‐SAC_340_	70	Holland et al. (2017)	Surface river 8.3066	Present study
DOC‐SAC_Ka310_			Surface river 1.518	Present study
DOC‐*R*_254/365_	3.45	Holland et al. (2017)	Surface river 5.7500	Present study
DOC‐*S*_R_			Surface river 0.9827	Present study
DOC‐FI	1.54	Holland et al. (2017)	Surface river 1.5906	Present study

*Note:* Data from the Rio Negro, Anavilhanas Archipelago and from the Rio Solimões near Manaquiri City at the nadir of the 2023 drought are compared to historical data recorded at a similar time of year. 2023 data come from the present study and from Braz‐Mota et al. (2025, this issue; and personal communication). Surface samples are collected in the top 10 cm. Deep samples are from depths of 150 cm or more. Abbreviations: ICMBio, Chico Mendes Institute for Biodiversity Conservation ranger station; LJ, Lake Janauacá; LP, Lake Prato; river, the main river.

^a^ From station S4 to S1 up the Prato channel.

^b^ Personal communication: Braz‐Mota (*Universidade Federal do Amazonas, Instituto Nacional de Pesquisas da Amazônia, Programa de Pós‐Graduação em Biologia de Água Doce e Pesca Interior, Manaus, Brazil*).

### 4.1 Habitat

The drought of 2023 was much more severe, and air temperatures were warmer than in 2013 and 2014 during an earlier study of the low‐water period in the Anavilhanas Archipelago of the Rio Negro (Espinoza et al., 2024; Johannsson et al., 2017). At the gauging station in Manaus, low water fell to 12.96 m in 2023 compared to 19.35 and 19.90 m in the two earlier years (https://portodemanaus.com.br/nivel-do-rio-negro/). Vertical and lake/forest habitats for fishes were much reduced at this time. No aquatic forest habitat remained along the river or in the lake basin, as had been observed in earlier surveys of the Rio Negro. The lake was much diminished, and its connection with the river had been lost. The river banks were high, and water depth in the river stem of the Rio Negro near the ranger station (ICMBios) was only 3 m. Large sand bars were exposed at the confluence of the channel to Lake Prato. They were not visible in 2013–2014. A similar study of ‘normal’ low‐water conditions has not been found for the Rio Solimões. However, again in 2023, the river banks rose high above the river, and Lake Janauacá was greatly reduced in area compared to 2019 (Zingel et al., 2026, this Special Issue).

During drought conditions, fishes are herded into smaller and smaller volumes of water by the receding of the lateral connections between lakes and rivers. Stressful abiotic conditions, such as higher temperatures and lower oxygen concentrations, act as strong environmental filters, forcing the movement of fish species into the main river stems (Freitas et al., 2010; Röpke et al., 2016). The biodiversity of the lakes and rivers changes seasonally. The Rio Negro normally develops a more diverse fish community, whereas the Rio Solimoes' fish community becomes less diverse (Saint‐Paul et al., 2000). Agasild et al. (2026, in this Special Issue) showed that seasonal water‐level fluctuations also lead to more distinct changes in the trophic dynamics of fish in Amazonian black‐water lakes (Lake Prato), where the community is pushed more towards planktivory. In white‐water lakes (Lake Janauacá) the trophic structure remains more diverse. The 2023 drought had progressed beyond that point as the lake waters were so low that only a small area remained. The diversity of the Lake Janauacá fish populations depended in part on the animal and plant communities associated with the extensive floating macrophyte islands and beds (Agasild et al., 2026 in this Special Issue). If these were no longer accessible, then one might expect the trophic diversity to also narrow in the Rio Solimões system.

Living in reduced water volumes can be stressful for fishes. The crowding of fishes results in social stress and less‐favourable feeding conditions, which lead to decreased growth associated with lower food intake, lower food conversion efficiency and changes in glycolytic and glycogenic metabolism (Santos et al., 2010; Trenzado et al., 2006). Coupled with crowding are the changes in physicochemical water quality conditions. The social, trophic and physical–chemical conditions caused by drought may act synergistically and likely explain why the condition factors and lengths of fish in both rivers were much reduced in 2023 compared to 2019 (Zingel et al., 2026, in this Special Issue). The black‐water system was more severely affected than the white‐water system (Zingel et al., 2026 in this Special Issue). This was likely due to differences in food web/fish community resilience between the two river systems (Agasild et al., 2026, in this Special Issue). Shifts in diet between high‐ and low‐water periods showed that niche breadth narrowed more in Lake Prato within the Anavilhanas Archipelago on the Rio Negro than in Lake Janauacá on the Rio Solimões, leading to greater competition within the Lake Prato fish community for food.

### 4.2 Temperature

Amazonian waters are expected to increase 2 to 7°C over the next century, assuming no further deforestation (IPCC, 2023). Studies in the past 8 years have revealed that a number of fish species are, on occasion, already close to their upper thermal limit (e.g., Campos et al., 2019; Jung et al., 2020). The fish community is under threat from increasing water temperatures and the associated decrease in dissolved oxygen. High temperatures and hypoxia, common in lakes during extreme drought periods, can compromise the hierarchical stability and metabolism of Amazonian fish such as *Apistogramma agassizii* (Steindachner, 1875) (Kochhann et al., 2015). Braz‐Mota et al. (2026, in this Special Issue) investigated the thermal and hypoxia tolerances of fishes from the black‐ and white‐water environments, specifically examining differences among taxonomic orders, to develop predictive information for the hundreds of fish species native to these waters. They also considered the thermal adaptability and vulnerability of the different fish orders in the two waters. Now and in the future, fish species may reach or be exceedingly close to their thermal maxima (Braz‐Mota et al., 2026; Campos et al., 2019).

Present information on temperature and oxygen concentrations in the Anavilhanas Archipelago remains limited even after the studies associated with the present expedition. Temperatures along the river transect, at 5 cm depth, ranged from 31.4 to 31.9°C in the early afternoon of 23 November. Temperatures at 150 cm depth, in the channels with river flow, were 29.1 to 31.1°C on 28 and 29 November. Diel measurements of temperature at 30 cm and 3.0 m depths provide a much more dynamic picture of the temperature habitat at the junction of the river and the channel leading to Lake Prato. High mid‐day peaks are a new phenomenon and averaged 34.0°C ± 0.97 (SD) (*N* = 6): range, 32.39–35.03°C (Braz‐Mota et al., 2026, in this Special Issue). Bottom temperatures fell over the period from 31.38 to 30.28°C. These diel patterns are similar to the daytime patterns observed in Lake Prato in 2013–2014 during normal low‐water conditions (Tables S1–S5 in Johannsson et al., 2017). Therefore, the Lake Preto fish species may be adapted to these temperature variations, though the riverine species may not.

The channel normally connecting the river to Lake Prato was no longer connected to the lake at the time of the expedition; consequently, water flow was dependent on ground water and expected to be very low. Only at S4, near the confluence with the river, there was some mixing as evidenced by the similarity of all measured variables at S4 with the river transect data (Figures 3a–e, 7a–f). Our thought is that the channel was isolated and behaving more like the lake as it dried out – with low oxygen observed at S2 and S3 (Figure 3c), shifts in DOC composition to autochthonous properties with lower SAC_340_ and higher FI (Figure 7c,f) and increases in pH (Figure 3d). Braz‐Mota et al. (2025, this issue) collected their diel oxygen measurements in this channel and observed no diel pattern and an average oxygen concentration of 3.06 mg O_2_ L^−1^. This concentration was not significantly different from the 2.96‐ and 3.26 mg O_2_ L^−1^ observed in the surface waters of channel stations S2 and S3 (Table S2b). Oxygen measurements in flowing environments were much higher. Surface measurements of oxygen concentration at 5 cm depth across the transect and into site S4 ranged from 5.14 ‐ to 6.23 mg O_2_ L^−1^ (Table S2b), similar to oxygen levels in Lake Prato in 2013 and 2014 (Johannsson et al., 2017; Table 2). Oxygen concentrations at 150 cm depth in the channels of the archipelago ranged from 6.14‐ to 7.05 mg O_2_ L^−1^ (Table S2a). There appear to be habitats of similar oxygen and temperature patterns, but they moved over years. These data and their comparisons lead to the conclusion that a three‐dimensional (horizontal, vertical and temporal) model/map of the archipelago is needed for key variables, such as temperature and oxygen, at low‐ and extremely low‐water conditions, to visualize the habitats available to fish both day and night. The recognition of similar habitat types should make this effort feasible. Habitats might be based on flow rate, depth and connectivity. With this model and general knowledge of the ecosystem, it should be possible to predict how habitats will change under different water‐level scenarios. Fish distribution needs to be linked to this model/map if we are serious about assessing the risks to and needs of fish species in the coming years.

### 4.3 High TSS in the Rio Negro

The Rio Negro has been known for its water transparency with Secchi depths of 90 to 100 cm, in spite of the dark colour of its waters (Val & Almeida‐Val, 1995; Johannsson et al., 2017; Table 2). Sediment in the water is composed of sand/silt and is usually <5 mg L^−1^ and always <11 mg L^−1^ (Crémazy et al., 2019; Marinho & Cremon, 2020). Yet during the drought of 2023, Secchi depth declined by half and TSS increased, ranging from 17.7 to 67.2 mg L^−1^ in surface waters (Figures 3a, 5a; Table 2). Increased suspended sediments reduce visibility in the water column, which can protect fishes from visual predators. Jönsson et al. (2011) observed that planktivorous fishes were more affected by loss of visual field than their filter‐feeding prey, *Daphnia commutate* G.O. Sars 1862.

Reduced visibility can reduce foraging efficiency by limiting visual‐based behaviours, such as feeding and mate selection. Reduced growth rates and altered gill structure (increases in ionocytes and thickness of lamellae and decreases in interlamellar space) have been observed (Montoya et al., 2024; Sutherland & Meyer, 2007). The level to which changes affect ionoregulation or respiration of fishes is less clear. Firth et al. (2024) observed loss of equilibrium at a reduced P_O2_ in eastern sand darter, and Crémazy et al. (2019) reported negative effects on sodium balance in cardinal tetra. Effects increased with the level of TSS, often at concentrations ≥100 mg L^−1^, although changes in gill structure were observed as low as 50 mg L^−1^ (Au et al., 2004). However, the types of sediment and particle sizes were not always reported with the studies. When sediment was described, it was more likely a clay/silt sediment. Sediments such as sand are rough and may abrade at lower TSS. If the SS size range 1.2–50 μm is the more dangerous, then the regions with the most abrasion effects would be at S3 and S4 (~40 mg L^−1^) and at S5 (~30 mg L^−1^). It is known that sediment in the water can impact filter‐feeding zooplankters, such as herbivorous cladocerans (Kirk, 1991). Cladoceran biomass in the surface samples (5 cm depth) was lowest at S3, S4 and S5 (Figure 6c). A significant, negative linear relationship was found between cladoceran biomass and the mass of the SS_1.2–50 μm_ size fraction (Figure 5d). Sediments, particularly the smaller size particles, reduce light penetration. The reduction in light penetration reduces the depth of the water column suitable for primary production. Fifty per cent reductions in Secchi depth were observed in 2023 compared to 2019 (Johannsson et al., 2017). Therefore, sediments negatively impact the planktonic food web in two ways: by reducing potential primary production and by reducing the filter‐feeding cladocerans that link primary production to the planktonic food web. Overall, the increase in TSS is not beneficial to fish well‐being due to its potential effects on the gills and its potential depression of primary production and cladocerans, which are favoured food for small fishes.

We suspect that the sediments were entering the water column due to the disturbance of the bottom substrates by fishes. It is unlikely that currents were responsible. Currents would have been weaker at this time due to lower water levels. Furthermore, the Rio Branco, which normally brings sand into the archipelago when it joins the Rio Negro, was also affected by the drought and would have had low flow rates. With the disturbance of the sediments, metals can enter the water column along with clay/sand. Metals in sediments can be many times more abundant than in the overlying water (e.g., Moulatlet et al., 2023). Most aqueous metal concentrations in these natural regions of the river are below the Brazilian COMANA guidelines (Küchler et al., 2000; Crémazy et al., 2016; tab. S1 in Johannsson et al., 2025). Whether sediment disturbance caused by drought leads to guideline exceedances is a question for future research.

### 4.4 Degradation of DOC in both rivers

DOC is integral to the structure and functioning of black‐water systems, and for their fish communities (Johannsson & Holland, 2024). DOC is a heterogeneous complex of hundreds of different molecules derived from the degradation of terrestrial and aquatic matter. Allochthonous DOC is derived from terrestrial plants and is characterized by lignin components, which give it its colour and high light‐absorptive characteristics (Ertel et al., 1986). Autochthonous DOC is produced by the degradation of organisms within the aquatic system (algae, bacteria, macrophytes, dead animals); and therefore, it does not have high lignin content. Degradation of DOC is a slow, continuous process. Sunlight photo‐oxidizes DOC with the production of carbon dioxide and, to a lesser extent, carbon monoxide. Bacteria and fungi attack the structures of the DOC, incorporating DOC carbon and nutrients into the microbial food web (Johannsson & Holland, 2024). In the end, usually in the ocean, only recalcitrant molecular structures are left. The state/quality of the DOC is assessed via a number of indices (Table S1). The DOC in the Rio Negro in 2023 was degraded compared to the DOC in December 2013 and 2014 (Johannsson et al., 2017; Table 2). Total ABS_250–550_ DOC^−1^ declined by approximately 50%, SUVA_254_ by 20%–45%, SAC_Ka310_ by 26% and SAC_340_ by 28%. *R*_254/365_ and FI did not change noticeably. A lack of change in mean molecular weight (*R*_254/365_) would suggest that *S*_R_ is also not changing. The Rio Negro DOC from the transect had lost aromaticity and capacity to produce ROS but had not changed in mean molecular weight. This means that the 2023 DOC may not have been as good at maintaining ionoregulation in fishes under conditions of low pH and low ion concentrations (Al‐Reasi et al., 2013; Galvez et al., 2008; Morris et al., 2024; Wood et al., 2003; Wood et al., 2011). However, the exact relationship between SAC_340_ and protection of ionoregulation still remains to be defined.

DOC absorbs light in accordance with its wavelength. UVB, a dangerous spectrum of light for all living organisms, is removed from the water by DOC more readily than UVA or visual wavelengths. The 2023 Rio Negro DOC removed 99% of UVB radiation in the top 5 cm. This expands the vertical range of algae, zooplankton and fishes in these black waters to the subsurface environment. In the Rio Solimões, DOC would remove ~50% of the UVB by 5 cm depth and 99% by ~38 m depth. The clay in the water column would reflect some of the UVB. UVB radiation may be one cause for the low numbers of zooplankton at 5‐cm‐deep waters of the Rio Solimões.

The Rio Solimões DOC observed in 2023 was autochthonous (high FI), composed of smaller molecules (high *R*_254/365_), had low aromaticity (SUVA_254_ and SAC_340_) and had very little capacity to produce reactive oxygen species (ROS) (Table 1). This DOC did not protect against an experimental spike of Cu, whereas the allochthonous DOC of the Rio Negro did protect the fishes (Morris et al., 2026, in this Special Issue). There is a complex relationship among DOCs, dissolved metals, osmoregulation and water quality. These relationships are gradually being unravelled by Galvez et al. (2008), Al‐Reasi et al. (2013), Sadauskas‐Henrique et al. (2021), Morris et al. (2024), Crémazy et al. (2022), Crémazy et al. (2026, in this Special Issue) and Sadauskas‐Henrique et al. (2026, in this Special Issue).

The DOC in the Rio Solimões was unusual in having very low aromaticity (SAC_340_ of 8.3) (Table 1), much lower than observed historically (Table 2). The FI (1.59) and *R*_254/365_ (5.75) indicated that the DOC was composed of small molecules of aquatic origin (bacterial/algal/macrophyte/animals) (Table 1). The high bacterial biomass (Figure 9a) suggests that a large degradation process had been occurring, which would be more characteristic of processes in the Várzea lakes, such as the degradation of macrophytes, dead fishes and other organisms as the lakes became shallower. We hypothesize that with the drought, lower and lower volumes of water were passed downstream from the upper reaches of the river. As water levels fell, the Várzea lakes, which border on the Rio Solimões, increased their flows into the river, altering the ratio of terrestrial to aquatic DOC in the river and greatly increased the DOC concentration.

### 4.5 Microbial food web in the Rio Solimões

The Gasol model indicated that the microbial food web in the Rio Solimoes was predator‐controlled (Figure 9b). Copepods are abundant in the Rio Solimões and thought to be the controlling predator (Agasild, personal communication). In Section 4.4, it was noted that the higher‐than‐normal concentration of Rio Solimões DOC and its autochthonous characteristics suggest more‐than‐normal degradation of in‐river material. This could help to explain the very large biomass of bacteria. Even so, the low abundances of HNF and ciliates negate any thought that the system was driven from the bottom up. This is confirmed by comparing the biomasses of the HNF and ciliates between the Rio Negro and the Rio Solimões. The Rio Negro is not a productive system compared to the Rio Solimões (Val & Almeida‐Val, 1995); however, the biomasses of the HNF and ciliates are much greater in the Rio Negro. Another sign that the Rio Negro is a system governed from the bottom up is the regular spacing between the tropic‐level biomasses (bacteria, HNF and ciliates). In predation‐dominated systems, the biomass between trophic levels often see‐saws, reflecting a trophic cascade. In a trophic cascade, one trophic layer (B) is heavily preyed upon by a predator (P), which decreases the biomass of layer (B) to such an extent that trophic layer (B) cannot control the biomass of its prey (Carpenter et al., 2001). This looks more like the situation in the Rio Solimões (Figure 9a). The bottom‐up system of control is the more efficient at delivering energy to the top of the food chain. The implication is that the energy flow through the planktonic food web in the Rio Solimões is reduced at this time, which could reduce the planktonic food resources of plankton‐eating fish (planktivores, detritivores and omnivores).

## 5 CONCLUSIONS

The increased fish mortality and reduced growth and condition factor of fishes observed during this extreme drought suggest that environmental and biotic factors, such as high temperature, oxygen reduction in some regions, reduced habitat volume, increased sediment load and altered food resources, can have synergistic negative effects on aquatic fauna. Drought causes multiple stressors, which can affect the fish community, resulting in both behavioural and physiological responses. Wood et al. (2026, in this Special Issue) demonstrated that variation in a range of environmental variables (pH, temperature, ammonia and partial pressures of oxygen and carbon dioxide) altered the transepithelial potential (TEP) across the gills of tambaqui (*Colossoma macropomum*). Alterations in TEP reflect adjustments at the gills, which enable the fish to maintain the ionic composition of its blood, but also may bear a metabolic costs (Zimmer & Perry, 2022). Shifting environmental conditions during these droughts may exact additional metabolic costs.

Given these observations, the need for ongoing investigations to understand the relationship between DOC, sediments and water quality at different spatial and temporal scales is evident. Additional monitoring can help identify emerging patterns and assess the impacts of drought on the fish community, contributing to conservation and management strategies for Amazonian aquatic ecosystems. Furthermore, analysing predation and fish metabolism under these extreme conditions may reveal unexpected adaptations and reinforce the importance of biological resilience in environments subject to significant environmental variations. The societal, ecological and physiological impacts observed during these droughts (Padilha et al., 2025; papers in this Special Issue) re‐enforce the need for monitoring and conservation actions to increase the resilience of the Amazon basin to environmental variability.

## AUTHOR CONTRIBUTIONS

Conceptualization: Ora E. Johannsson, Adalberto L. Val, Helen Agasild, Priit Zingel and Rafael M. Duarte. Data analysis: Ora E. Johannsson, Helen Agasild and Priit Zingel. Preparation of tables and figures: Ora E. Johannsson, Helen Agasild, Priit Zingel and Jhonatan Mota da Silva. Research: Ora E. Johannsson, Thiago L. Nascimento, Rafael M. Duarte, Helen Agasild, Priit Zingel, Gudrun de Boeck, Anne Cremazy and Carolyn Morris. Data interpretation, writing, reviewing: Ora E. Johannsson, Adalberto L. Val, H. A. Priit Zingel, Rafael M. Duarte, Gudrun de Boeck, Anne Cremazy, Carolyn Morris, Chris M. Wood and Thiago L. Nascimento, Overall supervision and logistics: Chris M. Wood and Adalberto L. Val.

## FUNDING INFORMATION

Funded in part by an NSERC (Canada) Discovery Grant (RGPIN‐2023‐03714) to Chris M. Wood. Chris M. Wood and Gudrun de Boeck received ADAPTA fellowships. Helen Agasild received Eesti Maaülikool, grant/award numbers: P210160PKKH, P220169PKKH, P190258PKKH; CNPq (465540/2014‐7); H2020 Spreading Excellence and Widening Participation, grant/award number: 951963; Eesti Teadusagentuur, grant/award number PUTJD1240. Both Helen Agasild and Priit Zingel received Instituto Nacional de Ciência e Tecnologia Centro de Estudos das Adaptações da Biota Aquática da Amazônia, grant/award numbers: FAPEAM (062.01187/2017) and 465540/2014‐7 (Priit Zingel only), other grants to Priit Zingel included Eesti Maaülikool, grant/award numbers: P190258PKKH, 465540/2014‐7; Horizon 2020 Framework Programme, grant/award number: 951963. Rafael M. Duarte was funded by a research fellowship through the INCT‐ ADAPTA II grant to Adalberto L. Val. Anne Cremazy was funded by Natural Sciences and Engineering Research Council of Canada (NSERC) Discovery Grant (RGPIN‐2019‐04400). Adalberto L. Val was funded by INCT ADAPTA (CNPq – Brazilian National Research Council – process no. 465540/2014‐7, CAPES – Coordination of Superior Level Staff Improvement – Finance Code 001 and FAPEAM – Amazonas State Research Foundation – process 062.01187/2017). Adalberto L. Val is also the recipient of a research fellowship from CNPq.

## CONFLICT OF INTEREST STATEMENT

None of the authors have a conflict of interest to disclose.

## Supporting information


**Data S1.** Supporting information.

## Data Availability

The data are available upon request of the corresponding author.
