# Supplementary material for: Amazonian drought of 2023: Environmental conditions relevant to fishes
Source: J Fish Biol. 2026 Jan 23;108(3):803–23. doi: 10.1111/jfb.70313 (PMC13122579; doi:10.1111/jfb.70313)
Supplement: Supplementary file 1 — Data S1. Supporting information. [file JFB-108-803-s001.docx]

Supplemental Data

**Amazonian Drought of 2023: Environmental Conditions**

**Relevant to Fish**

Ora E. Johannsson^a,b^, Thiago L. Nascimento^b^, Helen Agasild^c,b^, Priit Zingel^c,b^, Rafael M. Duarte^d,b^ , Gudrun de Boeck^e^, Anne Cremazy^f,b^, Jhonathan Mota da Silva^b^, Carolyn Morris^a,b^, Chris M. Wood^a,b^, Adalberto L. Val^b^.

^a^Department of Zoology, University of British Columbia, Vancouver, BC, V6T 1Z4 Canada

^b^Laboratory of Ecophysiology and Molecular Evolution, Brazilian National Institute for Research of the Amazon, INPA, Manaus, AM, Brazil

^c^ Department of Hydrobiology and Fisheries, Institute of Agricultural and Environmental Sciences, Estonian University of Life Sciences, Tartu, Estonia

^d^Biosciences Institute, São Paulo State University - UNESP, Coastal Campus, São Vicente, SP, Brazil

^e^ECOSPHERE, University of Antwerp, Campus Groenenborger, 2020 Antwerp, Belgium

^f^Institut National de la Recherche Scientifique, Centre Eau Terre Environnement, Québec, QC, G1K 9A9, Canada

*Corresponding author: Ora E. Johannsson

Email [johannss@zoology.ubc.ca](mailto:johannss@zoology.ubc.ca)

Telephone 1-778-772-1156

ORCID IDS and email addresses:

Ora E. Johannsson 0000-0002-7944-379X johannss@zoology.ubc.ca

Thiago Nascimento [thiagoluis@inpa.gov.br](mailto:thiagoluis@inpa.gov.br)

Helen Agasild 0000-0002-4926-9807 Helen.Agasild@emu.ee

Priit Zingel 0000-0003-1629-2063 priit.zingel@emu.ee

Raphael Duarte 0000-0001-5649-0692 [r.duarte@unesp.br](mailto:mduarte.rafa@gmail.com)

Gudrun De Boeck 0000-0003-0941-3488 gudrun.deboeck@uantwerpen.be

Anne Crémazy 0000-0002-0918-2336 anne.cremazy@inrs.ca

Jhonathan Mota da Silva 0000-0001-6907-0507 jhonatan.motabio@gmail.com

Carolyn Morris 0000-0002-7254-8433 [carolyn.morris19@gmail.com](mailto:carolyn.morris19@gmail.com)

Chris M. Wood 0000-0002-9542-2219 [woodcm@zoology.ubc.ca](mailto:woodcm@zoology.ubc.ca)

Adalberto Luis Val 0000-0002-3823-3868 [dalval.inpa@gmail.com](mailto:dalval.inpa@gmail.com)

| Suppl. Table S1. Absorbance and fluorescence indices used in the present study, their calculation, relevance and source. This table is a modification of the one presented in Johannsson et al. (2020). | | | | |
| --- | --- | --- | --- | --- |
|  | Name (units) | Calculation | Relevance | References |
| Absorbance  Indices | ABS_(250-550)_  (cm^2^.mg^-1^) | Sum of absorbance from 250 to 550 nm | Measure of total chromophore absorbance – and potential activity | Johannsson et al. 2017 |
|  | SAC_340_  (cm^2^.mg^-1^) | Specific absorbance at 340 nm: absorbance at 340 nm * 2.303. DOC^-1^ *1000) | Measure of aromaticity, protective potential of the DOC against some metals, and potential for direct biological effects on organisms | Curtis and Schindler, 1997  Wood et al. 2011  Al-Reasi et al. 2012  Al-Reasi et al. 2013 |
|  | SAC_(Ka310)_  (mg^-1^.m^-1^) or  (10 cm^2^.mg^-1^) | Specific absorbance at 310 nm:  2.303 * absorption at 310 nm/path length (0.01 m).DOC^-1^ | Measure of potential production of oxygen radicals per unit of DOC – used in degradation of DOC | Scully et al. 1996 Johannsson et al. 2017 |
|  | R_254/365_ | Ratio of absorbance at 254 nm to that at 365 nm | Index of the average molecular mass (size) within the DOC | Dahlen et al. 1996 |
|  | SUVA_254_  (mg^-1^.m^-1^) or  (10 cm^2^.mg^-1^) | Absorbance at 254 nm/pathlength (0.01 m).DOC ^-1^ | Measure of aromaticity, molecular weight, hydrophobic organic acid fraction | Abbt-Braun et al. 2004  Weishaar et al. 2003  Spencer et al. 2012  Chowdury 2013  Hansen et al. 2016 |
|  | Slope_275-295_  (cm^-1^.nm^-1^) | Slope of regression of the ln of absorbances from 275 to 295 nm | Index of molecular weight – correlated with weight of fulvic acid like compounds (but not humic acid-like compounds). Tracer of changes in DOC structure | Helms et al. 2008  Hayase and Tsubota, 1985  Carder et al. 1989 |
|  | Slope_350–400_  (cm^-1^.nm^-1^) | Slope of regression of the ln of absorbances from 350 to 400 nm | Reflects changes in high molecular weight compounds | Helms et al. 2008 |
|  | S_R_ | Ratio of  Slope_275-295_/ Slope_350–400_ | Reflects on the efficiency of degradation of large to small molecules in the DOC | Helms et al. 2008 |
| Fluorescence Index | FI | Ratio of the emission at 450 nm to 500 nm, 370 nm excitation | Relative measure of the autochthonous vs allochthonous DOC source | McKnight et al. 2001 |

Suppl. Table S2a. Sample locations and water quality data (PN = Rio Negro, PS = Rio Solimões). Measurements were made on the mornings of November 28^th^ and 29^th^ 2023 in the Rio Negro and in the morning of December 5^th^ in the Rio Solimões. Sites were located at depths in the vicinity of fish collections used for experiments documented within this Special Issue.

| Station ID | Latitude | Longitude | Time | | Depth  (cm) | Cond,  (µS cm^-1^) | Temp.  (^o^C) | Oxygen  (mg O_2_ l^-1^) | pH | Habitat |
| --- | --- | --- | --- | --- | --- | --- | --- | --- | --- | --- |
| **Rio Negro** | |  |  |  | |  |  |  |  |  |
| PN1 | 2^o^42’52.1”S | 60°44'42.3''W | 8:00 | <150 | | 16.5 | 29.1 | 4.75 | 4.5 | Lake -clear, shallow, no water movement |
| PN2 | 2^o^43’29.9”S | 60^o^44’57.6”W | 8:10 | 150 | | 11.6 | 29.9 | 6.14 | 5.0 | Connecting channel -clear, no water movement |
| PN3 | 2^o^43’29.9”S | 60^o^44’54.9”W | na | 150 | | 10.6 | 30.5 | 7.05 | 5.0 | Channel – clear, river flowing |
| PN4 | 2^o^44’31.0”S | 60^o^45’52.1”W | na | 150 | | 10.6 | 30.3 | 6.75 | 4.6 | Channel - clear, river flowing |
| PN5 | 2^o^43’13.3”S | 60^o^46’01.8”W | na | 150 | | 12.3 | 31.1 | 6.95 | 5.0 | Channel - clear, river flowing |
| PN6 | 2^o^40’21.3”S | 60^o^47’18.0”W | na | 150 | | 10.8 | 29.7 | 6.80 | 4.5 | Wide channel - clear, river flowing, |
| PN7 | 2^o^40’26.6”S | 60^o^48’33.4”W | na | 150 | | 13.8 | 29.1 | 7.82 | 3.9 | Wide channel - clear, river flowing |
| PN8 | 2^o^41’57.1”S | 60^o^46’27.7”W | na | 150 | | 10.9 | 30.6 | 6.00 | 4.9 | Channel - clear, river flowing |
| **Rio Solimões** | |  |  |  | |  |  |  |  |  |
| PS1 | 3^o^20’57.3”S | 60^o^11’47.2”W | 11:36 | <150 | | 142.8 | 32.2 | 5.96 | 7.0 | River – good flow |
| PS2 | 3^o^22’58.5”S | 60^o^13’43.5”W | 8:15 | <150 | | 74.1 | 26.9 | 5.11 | 5.5 | Lake – shallow, sluggish water, little main river influence |
| PS3 | 3^o^23’04.6”S | 60^o^13’56.5”W | 8:21 | <150 | | 72.1 | 26.9 | 5.30 | 5.1 | Lake – shallow, sluggish water, little main river influence |
| PS4 | 3^o^24’29.4”S | 60^o^15’18.4”W | 8:56 | 40 | | 65.6 | 26.4 | 3.40 | 5.1 | Lake – very shallow, sluggish thick water, little main river influence |
| PS5 | 3^o^21’29.6”S | 60^o^10’56.2”W | 10:42 | 150 | | 85.9 | 29.4 | 5.43 | 5.4 | Channel-paraná |
| PS6 | 3^o^20’51.6”S | 60^o^10’42.4”W | 11:01 | 150 | | 130.1 | 31.3 | 5.64 | 6.4 | River - good flow |
| PS7 | 3^o^20’32.3”S | 60^o^10’48.0”W | 11:20 | 150 | | 135.8 | 30.3 | 5.54 | 6.8 | River - good flow |
| PS8 | 3^o^21’30.8”S | 60^o^13’40.3”W | 11:45 | 150 | | 140.3 | 31.2 | 6.31 | 7.1 | River - good flow |

Suppl. Table S2b. Sample locations and water quality data. Samples were collected 5 cm below the surface between 9:45 a.m. and 2:45 p.m. November 23^rd^ in the Rio Negro and during the afternoon of November 30^th^ in the Rio Solimões. Cond = conductivity, Temp. = temperature, T. = transect, C. = connecting channel between lake and river, inbetween = inbetween the two ends of the channel or transect TSS is total suspended solids: 50 µm to 0.45 µm.

| Station ID | Latitude | Longitude | Habitat | Cond.  (µS cm^-1^) | Temp.  (^o^C) | Oxygen  (mg O_2_ l^-1^) | pH | Secchi (cm) | TSS  (mg l^-1^) | DOC  (mg C l^-1^) |
| --- | --- | --- | --- | --- | --- | --- | --- | --- | --- | --- |
| **Rio Negro** | |  |  |  |  |  |  |  |  |  |
| S1 | 2^o^43’09”S | 60^o^44’39”W | C. near lake | 40.2 | 29.9 | 5.16 | 5.1 | 33 | 55.9 | 10.4 |
| S2 | 2^o^43’09”S | 60^o^44’08”W | C. inbetween | 14.9 | 31.1 | 3.28 | 4.6 | 42 | 47.9 | 5.8 |
| S3 | 2^o^43’04”S | 60^o^44’58”W | C. inbetween | 14.4 | 31.2 | 2.76 | 4.5 | 32 | 67.2 | 8.2 |
| S4 | 2^o^43’05”S | 60^o^45’14”W | C. before river | 10.5 | 31.3 | 5.50 | 4.5 | 37 | 36.8 | 8.2 |
| S5 | 2^o^42’59”S | 60^o^45’27”W | T. NE shore | 9.6 | 31.4 | 6.18 | 4.5 | 38 | na | 8.1 |
| S6 | 2^o^43’01”S | 60^o^45’31”W | T. $¼$across T. | 9.3 | 31.6 | 6.21 | 4.4 | 52 | 17.3 | 10.3 |
| S7 | 2^o^43’03”S | 60^o^45’36”W | T. centre | 9.2 | 31.7 | 6.13 | 4.4 | 47 | 37.0 | 8.4 |
| S7* | 2^o^43’03”S | 60^o^45’36”W | T centre deep |  |  |  |  |  | 33.0 | 8.6 |
| S8 | 2^o^43’05”S | 60^o^45’40”W | T ¾ across T. | 9.3 | 31.6 | 6.10 | 4.4 | 42 | 24.3 | 9.3 |
| S9 | 2^o^43’07”S | 60^o^45’43”W | T. SW shore | 10.0 | 31.9 | 5.92 | 4.8 | 43 | 43.0 | 8.2 |
| **Rio Solimões** | |  |  |  |  |  |  |  |  |  |
| S10 | 3^o^20’51”S | 60^o^11’43”W | Northshore-open | 137.8 | 31.8 | 6.37 | 7.24 | 21 | 85.8 | 6.2 |
| S11 | 3^o^20’57”S | 60^o^12’01”W | Northshore-plants | 136.7 | 31.0 | 5.48 | 7.26 | 10 | 87.1 | 5.8 |
| S12 | 3^o^21’12”S | 60^o^12’15”W | T. ¼ across T. | 135.6 | 31.0 | 5.73 | 7.41 | 18 | 42.8 | 8.7 |
| S13 | 3^o^21’18”S | 60^o^12’21”W | T. midway | 134.4 | 30.9 | 5.65 | 7.25 | 17 | 123.7 | 7.9 |
| S14 | 3^o^21’26”S | 60^o^12’26”W | T. ¾ across T. | 134.9 | 31.1 | 5.50 | 7.42 | 18 | 77.7 | 5.9 |
| S15 | 3^o^21’34”S | 60^o^12’32”W | T. other side | 134.5 | 31.3 | 5.90 | 7.35 | 13 | 92.9 | 6.3 |
| S16 | 3^o^21’52”S | 60^o^12’24”W | C. 60 m in paraná |  | 31.5 | 4.37 |  | 5 |  | 9.2 |
| S17** | 3^o^21’08”S | 60^o^11’42”W | T. deep between S13and S14 | 136.4 | 30.9 | 5.32 | 7.24 |  |  | 6.4 |

*deep station in the Rio Negro transect: samples collected at 2.5 m, bottom = 3 m.

**station 17 sample was collected at 5 m depth. Bottom not determined.

Suppl. Table 3a. Abundance (individuals l^-1^) and distribution of species of metazoan zooplankton in surface waters (5-cm depth) at stations in the Anavilhanas Archipelago, Rio Negro, November 23^rd^ 2023. See Figure 1 and Table 1b for station locations and characteristics. Blanks indicate that the species was not found at that station. ‘cf’ indicates some uncertainty in the species identification.

| **Station/Group** | **S1** | **S2** | **S3** | **S4** | **S5** | **S6** | **S7** | **S8** | **S9** |
| --- | --- | --- | --- | --- | --- | --- | --- | --- | --- |
| **Copepods** |  |  |  |  |  |  |  |  |  |
| Calanoid copepodids | 0.1 |  |  |  |  | 0.4 |  | 0.1 | 0.2 |
| *Thermocyclops minutus* | 3.9 | 1.1 | 0.1 | 0.8 |  | 0.2 | 0.3 | 0.2 | 1.3 |
| Cyclopoid copepodids | 4 | 0.5 | 0.1 | 1.5 | 0.3 | 2.8 | 1.5 | 0.6 | 2.1 |
| Nauplii | 0.2 | 0.1 | 0.5 | 0.8 |  | 0.1 |  |  | 0.1 |
|  |  |  |  |  |  |  |  |  |  |
| **Cladocerans** |  |  |  |  |  |  |  |  |  |
| *Alona* sp. | 0.1 |  |  |  |  |  |  |  |  |
| *Bosmina hagmanni* | 2.5 | 4.3 | 0.8 | 0.9 | 4.3 | 11 | 5.8 | 6.7 | 25.3 |
| *Bosmina dubicen* | 0.1 |  |  |  |  |  |  |  |  |
| *Bosminopsis deitersi* | 1.5 | 0.4 |  | 4.9 | 1.1 | 4.9 | 2 | 1.9 | 15.1 |
| *Ceriodaphnia cornuta* |  |  |  | 0.1 |  | 0.1 |  |  |  |
| *Moina micrura* | 18.3 | 9.2 | 1.7 | 3 | 0.3 | 4.9 | 2.2 | 2.1 | 7.8 |
|  |  |  |  |  |  |  |  |  |  |
| **Rotifers** |  |  |  |  |  |  |  |  |  |
| *Asplanchna sieboldii* | 1.1 | 0.1 | 0.1 | 0.2 |  |  |  |  | 0.1 |
| *Brachionus* sp. | 6.8 | 0.5 | 0.3 | 0.2 |  |  |  |  |  |
| *Brachionus cf calyciflorus* |  | 0.2 | 0.2 | 1.8 |  | 0.5 |  | 0.7 | 4.4 |
| *Brachionus cf forficula* | 1.1 |  |  |  |  |  | 0.1 |  | 0.1 |
| *Brachionus falcatus* | 0.2 |  |  |  |  | 0.1 | 0.1 |  |  |
| *Filinia cf longiseta* | 0.2 |  | 0.2 |  |  |  |  |  | 0.1 |
| *Filinia* sp. |  |  | 0.2 |  |  |  |  | 0.1 |  |
| *Keratella americana* | 0.3 |  |  | 0.3 |  |  |  |  |  |
| *Lecane* sp*.* |  |  |  |  |  | 0.1 |  |  |  |
| *Mytilina* sp. | 0.1 |  |  |  |  |  |  |  |  |
| *Ploesoma hudsoni* |  |  |  | 0.1 |  |  |  |  |  |
| *Polyarthra* sp. |  |  | 0.1 | 0.1 |  |  |  |  |  |
| *Trichocerca similis* |  |  |  |  |  |  |  |  | 0.1 |
| *Trichocerca* sp. |  |  | 0.1 |  |  |  |  |  |  |

Suppl. Table 3b. Biomass (µg C.l^-1^) of metazoan zooplankton groups at 5-cm depth at stations in the Anavilhanas Archipelago, Rio Negro, November 23^rd^ 2023. Blanks indicate that the species was not found at that station. ‘cf’ indicates uncertainty in the species identification.

| **Station/Group** | **S1** | **S2** | **S3** | **S4** | **S5** | **S6** | **S7** | **S8** | **S9** |
| --- | --- | --- | --- | --- | --- | --- | --- | --- | --- |
| **Copepods** |  |  |  |  |  |  |  |  |  |
| Calanoid copepodids | 0.036 |  |  |  |  | 0.265 |  | 0.320 | 0.318 |
| *Thermocyclops minutus* | 1.470 | 0.448 | 0.089 | 0.347 |  | 0.073 | 0.101 | 0.068 | 0.479 |
| Cyclopoid copepodids | 0.935 | 0.116 | 0.020 | 0.202 | 0.077 | 0.497 | 0.247 | 0.087 | 0.423 |
| Nauplii | 0.051 | 0.029 | 0.064 | 0.078 |  | 0.025 |  |  | 0.019 |
| Total | 2.492 | 0.593 | 0.173 | 0.627 | 0.077 | 0.86 | 0.348 | 0.475 | 1.239 |
|  |  |  |  |  |  |  |  |  |  |
| **Cladocerans** |  |  |  |  |  |  |  |  |  |
| *Alona* sp. | 0.040 |  |  |  |  |  |  |  |  |
| *Bosmina hagmanni* | 0.519 | 0.687 | 0.176 | 0.176 | 0.931 | 2.516 | 1.173 | 1.888 | 5.389 |
| *Bosmina dubicen* | 0.015 |  |  |  |  |  |  |  |  |
| *Bosminopsis deitersi* | 0.340 | 0.045 |  | 0.587 | 0.140 | 0.694 | 0.360 | 0.288 | 2.204 |
| *Ceriodaphnia cornuta* |  |  |  | 0.022 |  | 0.038 |  |  |  |
| *Moina micrura* | 2.029 | 1.104 | 0.169 | 0.363 | 0.025 | 0.528 | 0.285 | 0.263 | 0.937 |
| Total | 2.943 | 1.836 | 0.345 | 1.148 | 1.096 | 3.776 | 1.818 | 2.439 | 8.530 |
|  |  |  |  |  |  |  |  |  |  |
| **Rotifers** |  |  |  |  |  |  |  |  |  |
| *Asplanchna sieboldii* | 0.148 | 0.013 | 0.013 | 0.027 |  |  |  |  | 0.013 |
| *Brachionus* sp. | 0.392 | 0.029 | 0.017 | 0.012 |  |  |  |  |  |
| *Brachionus cf calyciflorus* |  | 0.029 | 0.029 | 0.259 |  | 0.072 |  | 0.101 | 0.634 |
| *Brachionus cf forficula* | 0.063 |  |  |  |  |  | 0.006 |  | 0.006 |
| *Brachionus falcatus* | 0.019 |  |  |  |  | 0.010 | 0.010 |  |  |
| *Filinia cf longiseta* | 0.040 |  | 0.040 |  |  |  |  |  | 0.020 |
| *Filinia* sp*.* |  |  | 0.040 |  |  |  |  | 0.020 |  |
| *Keratella americana* | 0.016 |  |  | 0.016 |  |  |  |  |  |
| *Lecane* sp. |  |  |  |  |  | 0.007 |  |  |  |
| *Mytilina* sp. | 0.005 |  |  |  |  |  |  |  |  |
| *Ploesoma hudsoni* |  |  |  | 0.005 |  |  |  |  |  |
| *Polyarthra* sp. |  |  | 0.006 | 0.006 |  |  |  |  |  |
| *Trichocerca similis* |  |  |  |  |  |  |  |  | 0.005 |
| *Trichocerca* sp. |  |  | 0.005 |  |  |  |  |  |  |
| Total | 0.683 | 0.071 | 0.15 | 0.325 | 0 | 0.089 | 0.016 | 0.121 | 0.678 |

Suppl. Table 4a. Biomass (µg C l^-1^) of metazoan zooplankton groups at 5-cm depth at stations in Anavilhanas Archipelago, Rio Negro, November 23^rd^ 2023. 10 litres of water were filtered through a 0.50-µm mesh net. Samples were preserved in 70% alcohol and counted in their entirety. For species information, see Supplemental Tables 2a and b. For station locations see Fig. 1 and Suppl. Table S1b.

| **Location** | **Channel** | | | | **Transect** | | | | |
| --- | --- | --- | --- | --- | --- | --- | --- | --- | --- |
| **Station/Group** | **S1** | **S2** | **S3** | **S4** | **S5** | **S6** | **S7** | **S8** | **S9** |
| **Rotifera** | 0.683 | 0.071 | 0.15 | 0.325 | 0 | 0.089 | 0.016 | 0.121 | 0.678 |
|  |  |  |  |  |  |  |  |  |  |
| **Copepoda** | 2.492 | 0.593 | 0.173 | 0.627 | 0.077 | 0.86 | 0.348 | 0.475 | 1.239 |
|  |  |  |  |  |  |  |  |  |  |
| **Cladocera** | 2.943 | 1.836 | 0.345 | 1.148 | 1.096 | 3.776 | 1.818 | 2.439 | 8.530 |
|  |  |  |  |  |  |  |  |  |  |
| **Total** | 6.118 | 2.5 | 0.668 | 2.1 | 1.173 | 4.725 | 2.182 | 3.035 | 10.447 |

Suppl. Table 4b. Biomass and abundance of microbial loop components and total biomass of metazoan zooplankton. Biomass and abundance of bacteria, heterotrophic nanoflagellates (HNF) and ciliates (Ciliata) collected by Ruttner sampler from the two rivers late November and early December 2023. Metazooplankton were collected by Schindler Patalas traps. Subsamples were processed from a depth integrated, pooled sample. Metazozn biomasses (Agasild, pers. communication). Abund. = Abundance, Bact. = Bacteria.

| **River** |  | **Rio Solimões** | | |  | **Rio Negro** | | |
| --- | --- | --- | --- | --- | --- | --- | --- | --- |
| **Station** |  | **S12** | **S13** | **S15** |  | **S1** | **S2** | **S3** |
| **Bacteria** | Abund. x10^6^ cells ml^-1^ | 15.0 | 14.4 | 13.9 |  | 4.9 | 4.7 | 4.4 |
|  | Bact. biomass µg C l^-1^ | 870.8 | 797.2 | 722.7 |  | 215.4 | 197.8 | 175.6 |
|  |  |  |  |  |  |  |  |  |
| **HNF** | Abund. x10^3^ cells ml^-1^ | 1.2 | 1.3 | 1.5 |  | 9.4 | 8.2 | 7.3 |
|  | HNF biomass µg C l^-1^ | 24.5 | 27.1 | 28.1 |  | 172.2 | 140.7 | 111.8 |
|  |  |  |  |  |  |  |  |  |
| **Ciliata** | Abund. cells ml^-1^ | 6.7 | 8.2 | 10.7 |  | 17.4 | 21.2 | 23.5 |
|  | CILI biomass µg C l^-1^ | 26.7 | 34.5 | 49.0 |  | 59.5 | 76.0 | 87.8 |

Suppl. Figure S1. Spectral and fluorescence indices of DOC from the Rio Solimões. Samples were collected 5-cm below the surface in on November 30th 2023. Deep samples collected at S7 are presented as open circles. N = 1 (a) ABS_250-550_ (b) SUVA254, (c) SAC_340_, (d) SAC_Ka310_, (e) Slope Ratio(S_R_) and (f) Fluorescence Index.

1. (b)

Suppl. Figure S1. Index of mean molecular weight of the DOC molecules. R_254/365_ at (a) Rio Negro (RN) and (b) Rio Solimões (RS). Samples were collected 5-cm below the surface on November 23^rd^ 2023 (RN) and November 30^th^ 2023 (RS). Rio Negro data were analyzed by ANOVA followed by Tukey’s Multiple Comparison Tests. No significant differences were found. Only one or two replicates were measured in the RS; hence it could not be statistically analyzed. Note that S15 appears to have a lower ratio than the rest of the transect (see Table 4). A deep DOC sample collected at S7 is presented as open circles. N = 3.
